# Exosomes secreted by mesenchymal stem cells delay brain aging by upregulating SIRT1 expression

**DOI:** 10.1038/s41598-023-40543-5

**Published:** 2023-08-14

**Authors:** Xiaowen Zhang, Te Liu, Xuejia Hou, Zhongsheng Zhou, Fuqiang Zhang, He Ma, Xiaodong Wu, Jinlan Jiang

**Affiliations:** 1 Scientific Research Center, China-Japan Union Hospital of Jilin University, Changchun, 130000 Jilin China; 2 Yibin Jilin University Research Institute, Jilin University, Yibin, 644000 Sichuan China; 3 Department of Orthopaedics, China-Japan Union Hospital of Jilin University, Changchun, 130000 Jilin China

**Keywords:** Neuroscience, Stem cells, Medical research, Neurology

## Abstract

The increase in the aging population has seriously affected our society. Neurodegenerative diseases caused by aging of the brain significantly impact the normal life of the elderly, and delaying brain aging is currently the focus of research. SIRT1 is a viable therapeutic target, and there is mounting evidence that it plays a significant role in the aging process. Mesenchymal stem cell-derived exosomes (MSC-Exos) have gained widespread interest as nanotherapeutic agents because of their ability to be injected at high doses to reduce the immune response. The present study focused on the ameliorative effect of MSC-Exos on aging mice and the potential mechanisms of this effect on cognitive impairment and brain aging. In this study, we first tested the neuroprotective effects of MSC-Exos in vitro on H_2_O_2_-induced oxidative damage in BV2 cells. An in vivo SAMP8 rapid senescence mouse model showed that MSC-Exos significantly increased SIRT1 gene expression in senescent mice. In addition, MSC-Exos also had an anti-apoptotic effect and reduced oxidative stress in the brains of SAMP8 senescent mice. In conclusion, MSC-Exos may exert neuroprotective effects and help prevent brain senescence in SAMP8 mice by activating the SIRT1 signaling pathway.

## Introduction

Aging is the progressive loss of cellular homeostasis that leads to an overall decline in the adaptability of an organism^1^. As the human population lives longer, many age-related diseases become more prevalent, placing an increasing burden on health and social systems. Understanding how and why we age is vital to finding ways to live longer and healthier lives^2^. A stable type of cell cycle arrest brought on by telomere shortening or cellular stress is called cellular senescence^3^. Senescent cells tend to build up in aging tissues, and their removal can prevent old age-associated disease from occurring^4^. This shows a crucial connection between biologic aging and cellular aging. As people age, cognitive impairment follows neurodegeneration, which lowers the quality of life for the individual^5^.

Mesenchymal stem cells (MSCs) have the capacity for both self-renewal and cell type differentiation^6^. An increasing number of studies have shown that the therapeutic function of MSCs is mainly attributed to the paracrine effect mediated by MSC secretory factors, with studies reporting that exosomes play a major role in this effect^7,8^. Exosomes are extracellular vesicles with a diameter of 30–200 nm^9^, and their activity can be maintained by storage at − 80 °C. Exosomes can transport fats, proteins, enzymes, polynucleotides, and other forms of RNA, including microRNA, messenger RNA, and non-coding RNA^10,11^. A number of protein markers, including TSG101, CD9, and Alix, are also present in exosomes. Exosomes have a better safety profile than the MSCs that secrete them, are easier to preserve, and do not lose their function^12^. Therefore, exosomes have emerged as a promising cell-free treatment for treating human sickness, and their properties have attracted the attention of researchers.

Sirtuin 1–7 are the seven siblings of the Sirtuin relatives (SIRT1-7)^13^. Under some circumstances, SIRT1 translocates from the nucleus to the cytoplasmic matrix^14^. SIRT1 participates in a variety of cellular metabolic processes, including inflammatory, oxidative stress, apoptotic, and the development of tumors^15,16^. SIRT1 antagonizes hydrogen peroxide (H_2_O_2_)-induced premature senescence by negatively regulating p53 through the deacetylation of Lys-373, Lys-382, and Lys-320^17^. It has been demonstrated that neurons in the hippocampus, a crucial component for memories and learning, produce SIRT1^18–20^. SIRT1 is the most evolutionarily conserved sirtuin in mammals. It is essential to a variety of biological functions, including the regulation of metabolism, stress response, genomic stability, and ultimately in aging^21^. It has also been suggested that SIRT1, which regulates cellular energy and redox state and possesses NAD + -dependent protein acetylation activity, plays a factor in longevity because it fends off oxidative stress as we age and lengthens life.

Therefore, we suggest that reducing oxidative stress and apoptosis via the SIRT1 signaling pathway could be a promising strategy for the prevention and treatment of age-related illnesses. In order to provide a thorough theoretical foundation for the practical application of MSC-Exos in the treatment of age-related diseases, the study's objectives included evaluating the effects of MSC-Exos on cognitive deficits and brain damage in SAMP8 senescent mice and validating our hypothesis by evaluating the protein and gene expression associated with the SIRT1 signaling pathway.

## Results

### Characterization of mesenchymal stem cells and exosomes

**Figure 1:**
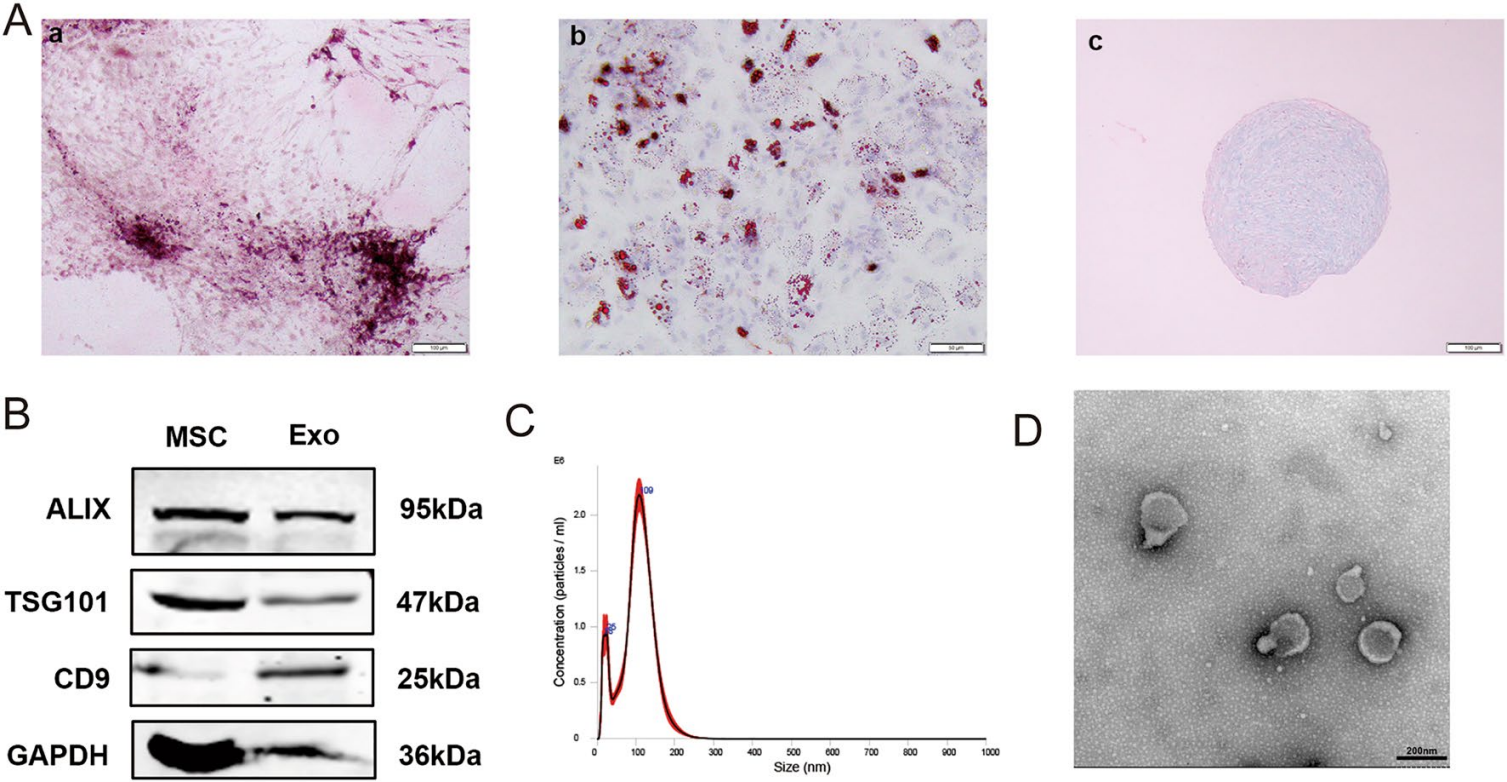
Identification of MSCs exosomes. (**A**) The results of experiments in figures a, b, c show that MSCs can be successfully induced into osteoblasts, lipoblasts, and chondrocytes. (**B**) Western blotting of protein expression in exosomes and MSCs. (**C**) Size distribution of MSC-Exo isolated by nanoparticle tracking analysis (NTA). (**D**) Representative transmission electron microscopy (TEM) images presenting the morphology of MSC-Exos.

First, we obtained and characterized MSCs that could differentiate into adipocytes, osteoblasts, and chondrogenic cells under specific culture conditions (Fig. 1A). The standard exosome markers CD81, TSG101, and Alix, which are typically on the MSC surface, were present in MSC-Exos that were obtained after a series of ultracentrifugations. Additionally, TEM and nanoparticle tracking analysis (NTA) were used to study the morphology and size distribution of exosomes. The findings demonstrated that the vesicles had the well-known bilayer "rim of a cup" structure and granule form, and that the bulk of the exosomes from MSCs had the characteristic exosome diameter (116 ± 2.6 nm) (Fig. 1B–D).

### Concentration mapping of H_2_O_2_ on the induction of BV2 cell senescence

**Figure 2:**
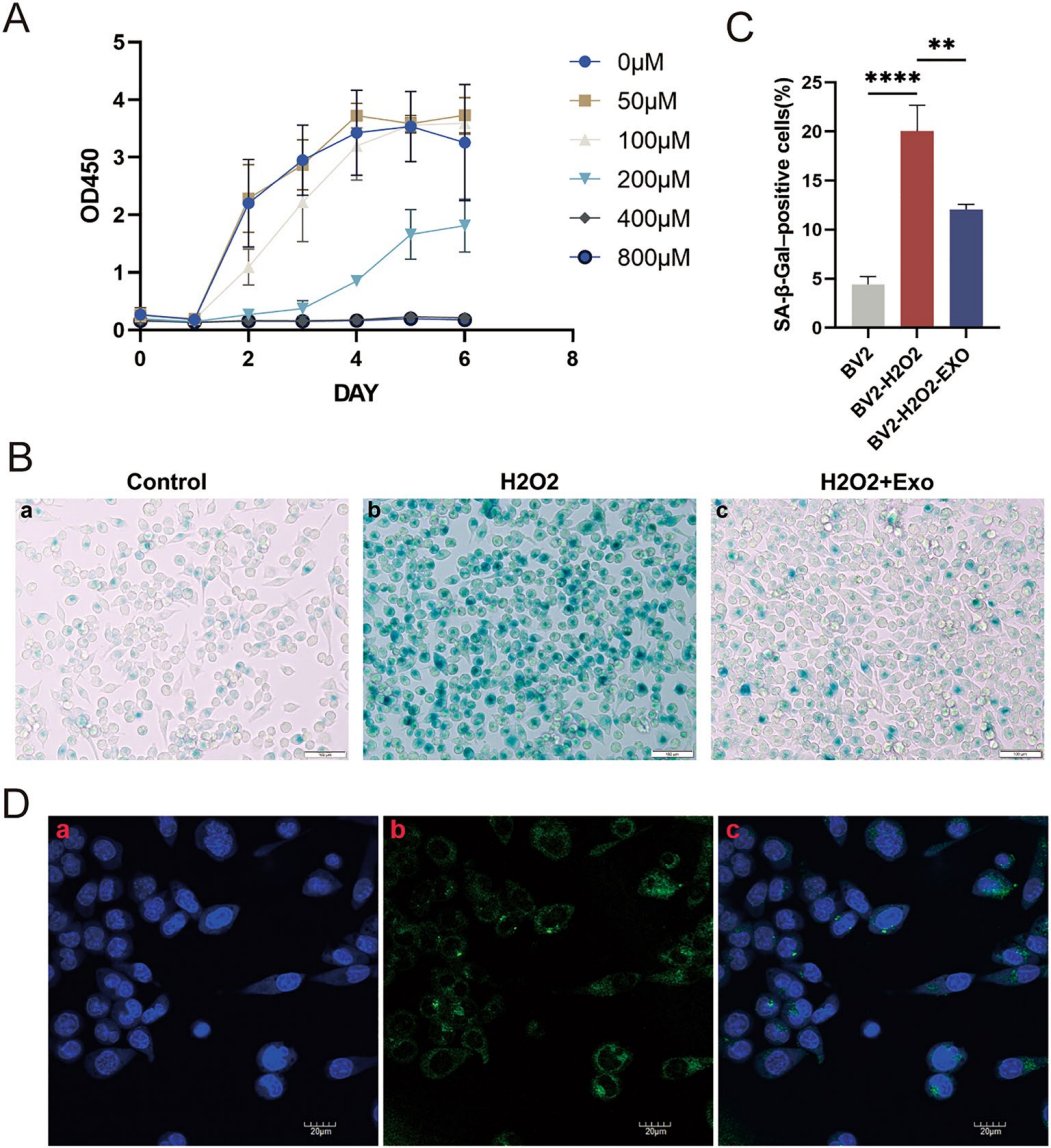
(**A**) Concentration of H_2_O_2_ on the induction of senescence in BV2 cells. (**B**) β-galactosidase staining, (a, b, c) control, H_2_O_2_-induced, and MSC-Exo treatment groups. (**C**) Quantitative analysis of senescent cell density by β-galactosidase staining. Data are expressed as mean ± SD of each group (n = 6). **P < 0.01, ****P < 0.0001. (**D**) Confocal microscopy image showing incorporation of MSC-Exos in BV2 cells; blue indicates DAPI staining of the nucleus, green indicates DiO-labelled MSC-Exos.

To determine the appropriate H_2_O_2_ concentration for the induction of senescence in BV2 cells, CCK8 assays were performed at concentrations of 0, 50, 100, 200, 400, and 800 μM H_2_O_2_ (Fig. 2A). Finally, 200 μM H_2_O_2_ was found to be the optimal concentration for inducing senescence.

### Effect of MSC-Exos on H_2_O_2_-induced senescence in BV2 cells

To investigate and confirm whether MSC-Exos alleviate H_2_O_2_-induced senescence in BV2 cells, senescent cells were detected using a β-galactosidase staining kit. The results of β-galactosidase staining showed that exposure to H_2_O_2_ resulted in significant cellular senescence compared with the control. However, MSC-Exo treatment significantly reduced the rate of cellular senescence (Fig. 2B,C). These results suggest that H_2_O_2_-induced senescence in BV2 cells can be effectively reversed by treatment with MSC-Exos.

### Tracking of MSC-Exos in BV2 cells

To assess the effect of MSC-Exos on BV2 senescence, we first confirmed whether MSC-Exos could be incorporated into BV2 cells. BV2 cells were labeled with DAPI (blue) and exosomes with DiO (green). The fluorescence results showed that the primary localization of green fluorescent dye (DiO)-labeled MSC-Exos in the perinuclear region of BV2 cells after 18 h of incubation indicated that MSC-Exos could enter the cells and modulate their biological behavior (Fig. 2D).

### MSC-Exos promote the proliferation and migration of BV2 cells

**Figure 3:**
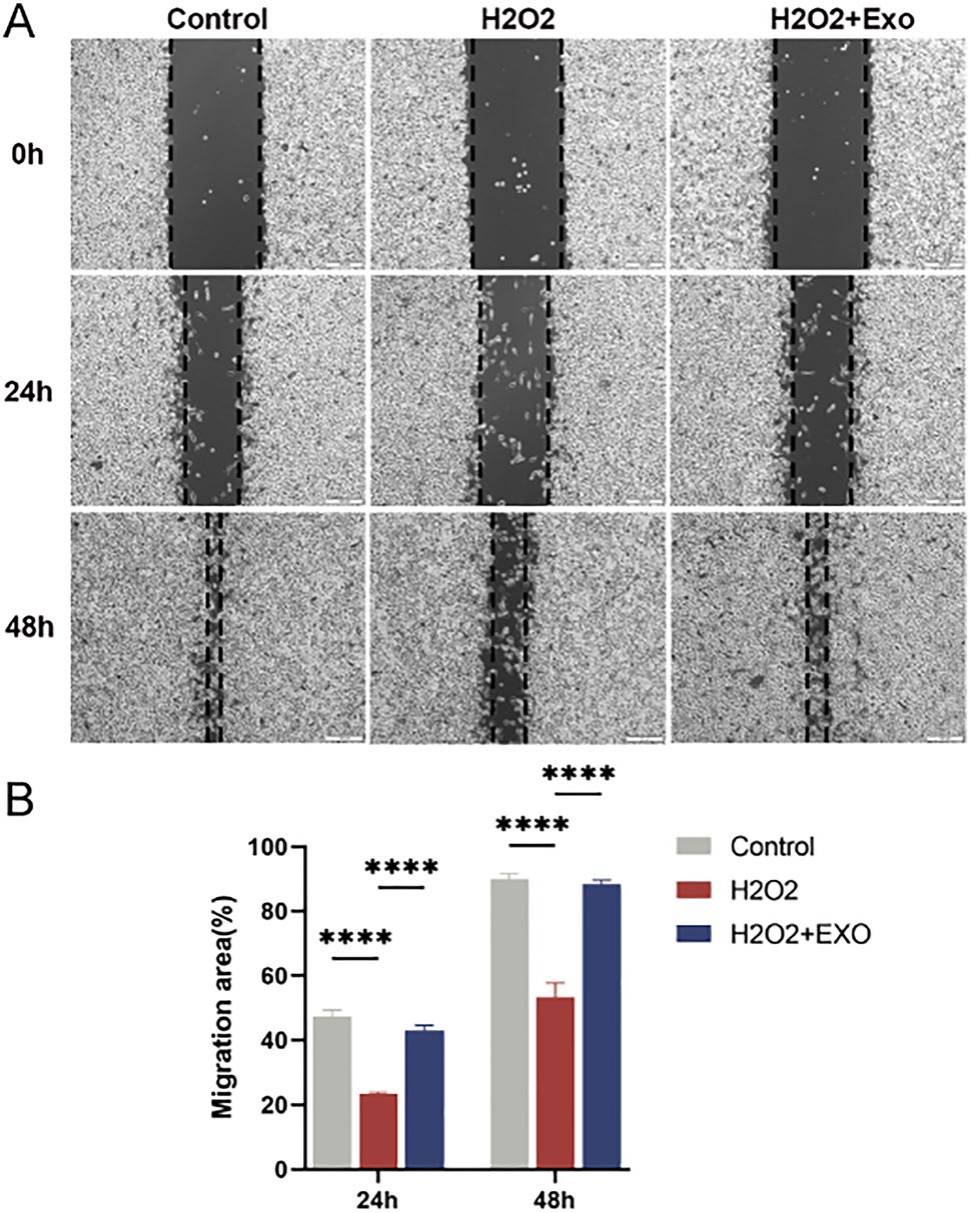
(**A**) Effect of MSC-Exos on proliferation and migration of BV2 cells. Light microscopic images of BV2 cells after 24 or 48 h of growth in fresh serum-free medium containing 50 μg/mL Exos. (**B**) Quantitative analysis of the rate of cell migration for the scratch assay. Data are expressed as mean ± SD of each group (n = 3). ****P < 0.0001.

To assess whether MSC-Exos promoted the proliferation and migratory capacity of BV2 cells, we performed a scratch wound assay. The findings revealed that the proliferation and migration rates of treated BV2 cells were significantly reduced in H_2_O_2_-treated BV2 cells compared to the control (Fig. 3A), while the proliferation and migration of BV2 cells were significantly enhanced with MSC-Exos (Fig. 3B).

### Histopathological observations

**Figure 4:**
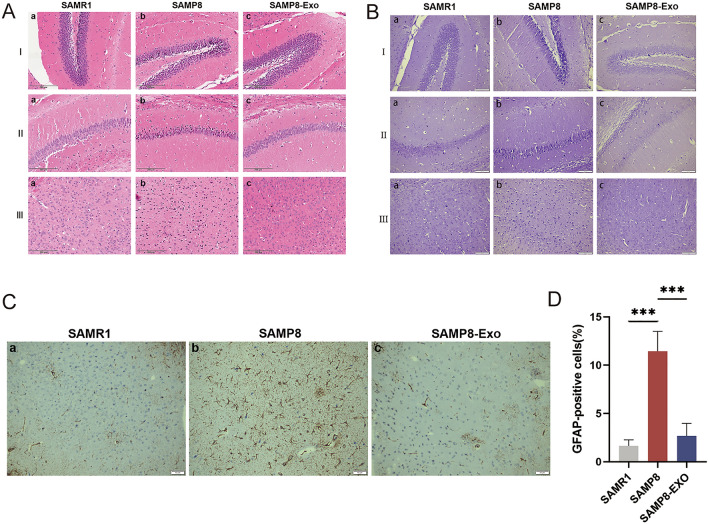
(**A**) H&E staining I: Histopathological characteristics of the dentate gyrus (DG) region of the hippocampus. a: SAMR1 control group; b: SAMP8 model group; c: MSC-Exo treatment group. II: Histological characteristics of the CA1 region of the hippocampus. a: SAMR1 control group; b: SAMP8 model group; c: MSC-Exo treatment group. III: Histopathological features of hippocampal cortical neurons. a: SAMR1 control group; b: SAMP8 model group; c: MSC-Exo treatment group. (**B**) Nissl staining I: histopathological features of the DG region of the hippocampus. a: SAMR1 control group; b: SAMP8 model group; c: MSC-Exo treatment group. II: Histological characteristics of the CA1 region of the hippocampus. a: SAMR1 control group; b: SAMP8 model group; c: MSC-Exo treatment group. III: Histopathological characteristics of hippocampal cortical neurons. a: SAMR1 control group; b: SAMP8 model group; c: MSC-Exo treatment group. (**C**) GFAP immunohistochemical staining. a: SAMR1 control group; b: SAMP8 model group; c: MSC-Exo treatment group. (**D**) GFAP-positive cell density. Data are expressed as mean ± SD of each group (n = 12). ***P < 0.001.

Regular features of cortical neurons in the control group were revealed by H&E staining. The SAMP8 model group showed shrinkage of apoptotic cells in different parts of the brain. Neuronal nuclei were laterally shifted and had dark staining. Additionally, there were fewer neurons in the cerebral cortex, which was significantly improved after MSC-Exos treatment (Fig. 4A). In addition, the neuronal cells in the CA1 and dentate gyrus (DG) regions of the hippocampus of control mice had clear morphology, regular structure, and tight intercellular arrangement. Compared with the model group, the MSC-Exos group showed a significant improvement in the morphology, structure, and arrangement of hippocampal neuronal cells and a significant reduction in the proportion of degenerated cells. In the Nissl staining assay, severe damage or loss of neurons was observed in the CA1 and DG regions of hippocampal tissue (Fig. 4B). The results showed a substantial decrease in the number of hippocampal neurons in SAMP8 mice, in addition to a substantial decrease in the number of Nissl bodies in SAMP8 senescent mice. On the contrary, the quantity of neurons in the hippocamp bodies treated with MSC-Exos was significantly restored. These findings imply that MSC-Exo therapy significantly ameliorates the hippocampal damage induced in SAMP8 mice. In addition, immunohistochemical findings imply that GFAP expression was significantly downregulated in the hippocampus of SAMP8 mice after MSC-Exo treatment (Fig. 4C,D).

### Effect of MSC-Exos on behavioral experiments in mice

#### Contextual fear-conditioning test

**Figure 5:**
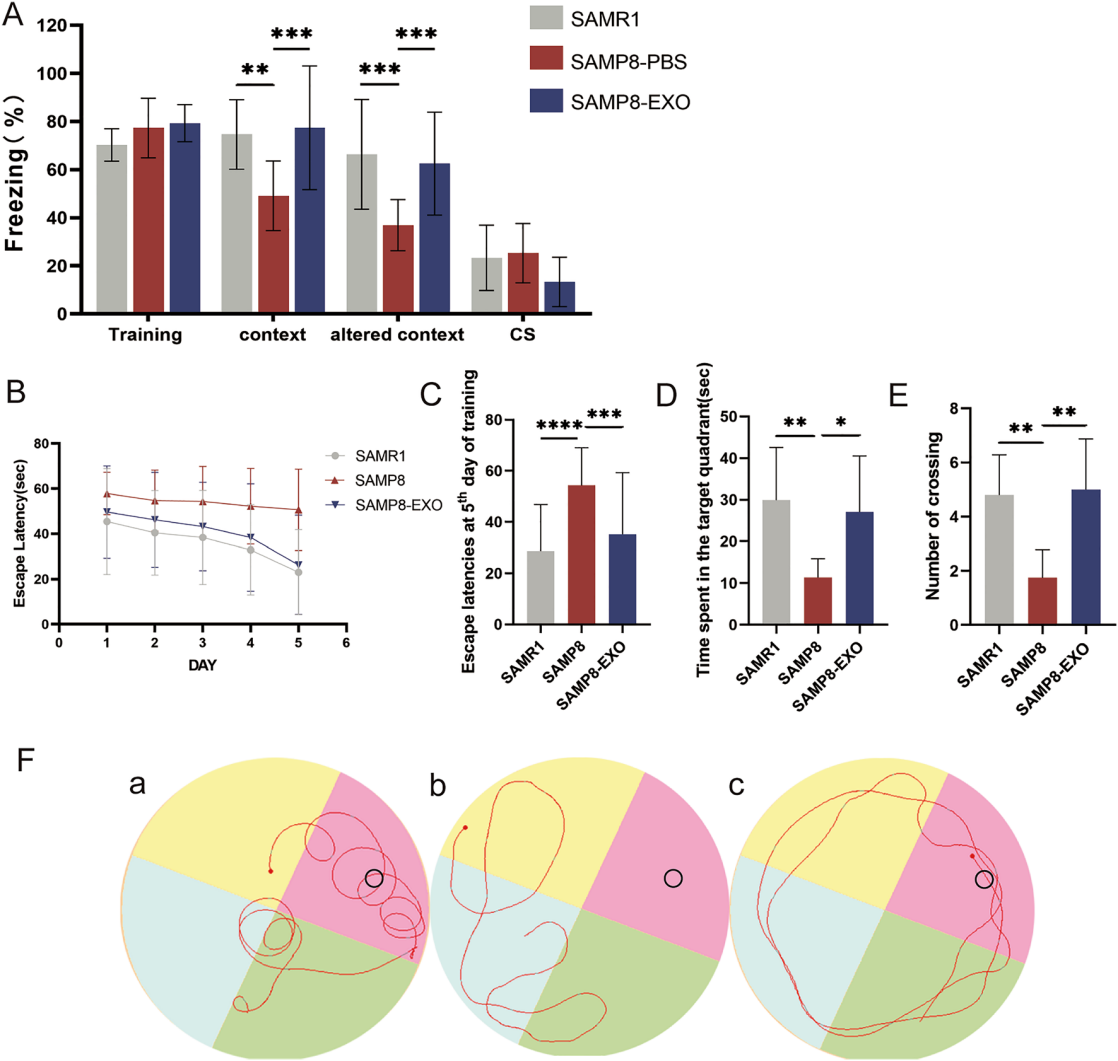
Contextual fear-conditioning test. (**A**) Freezing time in mice after stimulation (**B**) Escape latencies of the training trial. (**C**) Escape latencies at the 5th day of the training trial. (**D**) Time spent in the target quadrant during the probe test. (**E**) Number of crossings into the former location of the platform during the probe test. (**F**) Representative traces on the last day of training session and probe test. a: SAMR1 control group; b: SAMP8 model group; c: MSC-Exo treatment group. *P < 0.5, **P < 0.01, ***P < 0.001.

Next, we evaluated the learning memory capacity of MSC-Exos in SAMP8 senescent mice in a contextual fear conditioning experiment (Fig. 5A). The results from the association test and the freezing of the altered association test showed that the time to freezing in SAMP8 senescent mice was lower than that in the SAMR1 control group, while the time to freezing in MSC-Exo-treated mice was prolonged. This indicated a significant improvement in the memory capacity of the treated mice.

#### Morris water maze test

The Morris water maze was utilized in this work to examine the effects of MSC-Exos on spatial learning and memory in SAMP8 aging model mice. The delay in getting to the platform was significantly longer in SAMP8 mice, and MSC-Exo treatment significantly reduced escape latency during training (Fig. 5B,C). In probe-free experiments, SAMP8 senescent mice entered the pre-platform position less frequently and spent less time in the intended region compared to control SAMR1 mice (Fig. 5D,E). Interestingly, MSC-Exo-treated SAMP8 mice showed significant reversal of these adverse conditions (Fig. 5F). These findings suggest that MSC-Exos can restore memory and learning deficits in SAMP8 senescent mice.

### MSC-Exos inhibit oxidative stress in the brains of SAMP8 senescent mice

**Figure 6:**
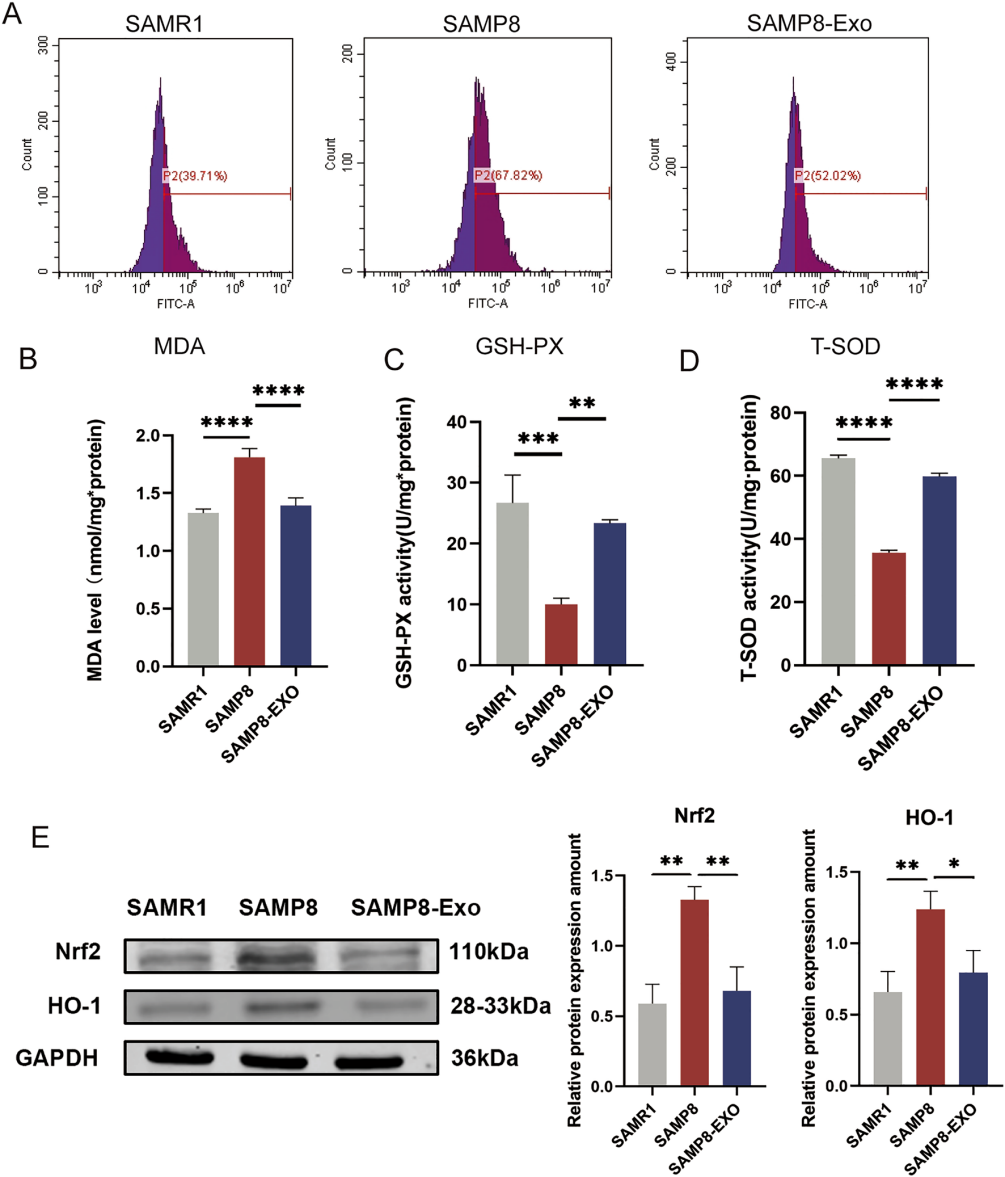
MSC-Exos attenuate the increase in oxidative stress in the brains of SAMP8 senescent mice. (**A**) Flow diagram of reactive oxygen species (ROS) levels in single cell suspensions of mouse brain. (**B**) Histogram representing malondialdehyde (MDA) levels in mouse brain homogenates. (**C**) Representative histograms showing glutathione peroxidase (GSH-Px) levels in mouse brain homogenates. (**D**) Representative histograms showing total superoxide dismutase (T-SOD) levels in mouse brain homogenates. (**E**) Results of protein blots for nuclear factor red lineage 2-related factor 2 (Nrf2) and heme oxygenase-1 (HO-1), and histograms of quantitative protein analysis. GAPDH was used as a loading control. *P < 0.5, **P < 0.01, ***P < 0.001, ****P < 0.0001.

First, we measured oxidative stress in the brains of SAMP8 senescent mice by measuring ROS. To this end, we performed ROS measurements on mouse brain tissue in all treatment groups (Fig. 6A). The ROS results showed that SAMP8 senescent mice showed dramatically raised oxidative stress in the hippocampus of the mouse brain. However, MSC-Exo treatment ameliorated the oxidative stress in the hippocampus. In addition, we examined GSH-Px, T-SOD, and MDA levels (Fig. 6B). These assays showed that MSC-Exo-treated SAMP8 mice had significantly increased GSH-Px and T-SOD levels and significantly decreased MDA levels in their brain tissue compared to control SAMR1 mice (Fig. 6C,D). Similarly, the expression of Nrf2 and HO-1 proteins was significantly inhibited in the hippocampus of SAMP8 senescent mice (Fig. 6E). Western blotting results showed that MSC-Exo treatment dramatically raised the expression of Nrf2 and HO-1 proteins in the hippocampus.

### Protective effect of MSC-Exos on brain senescence in SAMP8 mice through modulation of the SIRT1/p53 signaling pathway

**Figure 7:**
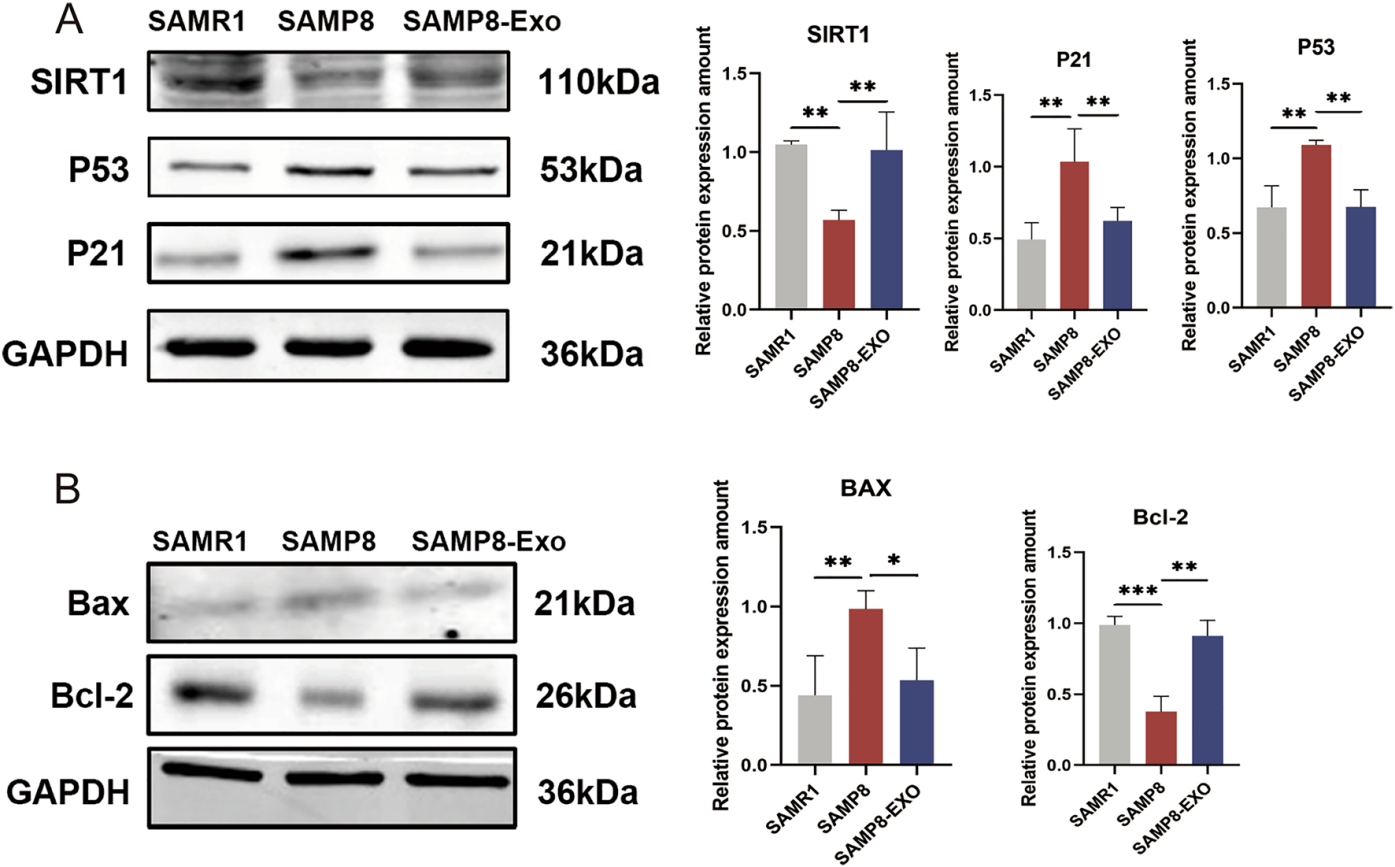
(**A**) Effect of MSC-Exos on SIRT1/p53 signaling in hippocampal tissues of SAMP8 senescent mice. (**B**) MSC-Exos attenuated excessive apoptosis in hippocampal tissues of SAMP8 senescent mice. Data are expressed as mean ± SD of each group (n = 12). *P < 0.5, **P < 0.01, ***P < 0.001.

We further examined the impact of MSC-Exos on SIRT1 expression using western blot analysis to assess if MSC-Exo therapy in brain aging is uniquely regulated through SIRT1 signaling. The results showed that SIRT1 protein expression was significantly reduced in SAMP8 senescent mice and that MSC-Exos significantly reversed the decrease in SIRT1 expression. We also investigated whether changes in p53 expression were regulated by MSC-Exos by western blotting to detect the protein expression of p53 and p21. As shown (Fig. 7A), the expression of p53 and p21 was upregulated in SAMP8 senescent mice, and MSC-Exo treatment significantly alleviated these changes.

Apoptosis-related proteins including Bcl-2 and Bax were also found. According to the figure (Fig. 7B), the results showed that Bax expression was significantly elevated in the hippocampus of SAMP8 senescent mice, and Bcl-2 expression was significantly decreased compared to that in the normal group. In contrast, Bax expression was significantly reduced, and Bcl-2 expression was significantly upregulated in the MSC-Exo-treated group. These data suggest that MSC-Exos exert anti-apoptotic effects and protect senescent mice from cell damage.

## Discussion

Aging is a progressive process characterized by cumulative degenerative damage associated with a range of morphological and alterations in behavior, such as motor and cognitive function that are associated with neurodegenerative impairment, ultimately leading to the death of the organism^22^. Ageing is evidently a complex process, and researchers have proposed that a number of mechanisms are involved, including oxidative stress, inflammation, and apoptosis. Establishing an animal model of aging is a crucial step in evaluating a drug's anti-aging abilities. The rapidly aging mouse (SAMP8) is an ideal model for studying aging and dementia, as it shows age-related learning and memory deficits^23^. The majority of aging's pathogenesis-related traits are manifested in SAMP8 animals, including oxidative stress, inflammation, and neuroapoptosis, as well as abnormal anti-aging factor expression. Six-month-old SAMP8 mice have been reported to exhibit impaired memory, early cognitive dysfunction, and neuronal damage contrasted to SAMR1 mice who are age-matched^24^. In addition, we observed increased oxidative stress and apoptosis in the brains of 6-month-old SAMP8 mice.

Mesenchymal stem cell-derived exosomes (MSC-Exos) can reduce oxidative stress and apoptosis in neural tissue, thereby slowing brain aging. In the current research, we assessed the neuroprotective effects of MSC-Exos. We used H_2_O_2_ to induce senescence in BV2 cells in vitro, and as can be seen in Fig. 2B, exosome treatment significantly reduced the number of senescent cells. As can be seen in Fig. 3, exosome treatment significantly promoted cell proliferation and migration ability. We assessed the effect of MSC-Exos on spatial learning and memory function by using the Morris water maze experiment and contextual fear conditioning experiment in SAMP8 experimental mice compared with SAMR1 control mice. In addition, we investigated the effects of MSC-Exos on hippocampal neuronal hyperapoptosis and ROS-mediated oxidative stress. We have also shown that the *SIRT1* gene is inactivated in SAMP8 aged mice and that exosomes secreted by MSCs can reactivate it. To the best of our knowledge, this study shows for the first time that (1) MSC-Exo treatment ameliorates the BV2 cell senescence induced by H_2_O_2_ in vitro, (2) MSC-Exos ameliorate cognitive deficits in SAMP8 senescent mice, and (3) MSC-Exos have an inhibitory effect on oxidative stress and apoptosis in neuronal cells of SAMP8 senescent mice.

The presence of ROS is both injurious and beneficial; at low concentrations, it plays an indispensable role in various physiological processes. However, excessive ROS production in tissues and cells can have toxic effects on the brain. There have been claims that oxidative stress plays a crucial part in the pro-inflammatory response and is one of the key causes of neuronal apoptosis, which is connected to aging naturally^25^. An essential transcription factor and oxidative stress sensor, Nrf2, shields cells against invaders and oxidative harm. Previous research has demonstrated that Nrf2 is primarily found in the cytoplasm under physiological circumstances; conversely, Nrf2 increases the translation of anti-oxidative stress proteins, such as HO-1, when ROS levels are excessive^26^. As can be seen in Fig. 6E, Nrf2 and its downstream gene HO-1 are expressed at higher levels in SAMP8 mice, pointing to potential roles for these proteins in the aging brain. In contrast, MSC-Exo treatment significantly ameliorated the elevated levels of oxidative stress, as revealed by the reduction in ROS levels and changes in GSH-Px (Fig. 6C), T-SOD (Fig. 6D), and MDA (Fig. 6B) in MSC-Exo-treated mice. Furthermore, reversal of Nrf2 and HO-1 protein expression levels in MSC-Exo-treated SAMP8 mice confirmed that MSC-Exo treatment is an effective antioxidant against oxidative stress in the brains of SAMP8 mice.

Apoptosis is widely known as programmed cell death, and it does not cause an inflammatory response^27^. Apoptosis causes distinctive morphological and metabolic alterations in cells. Involvement of programmed cell death is crucial for many physiological and pathological processes and is a complex process involving multiple genes^28^. Proteins of the Bcl-2 family are major regulators of apoptosis, leading to the activation of the effector member Bax^29^. Bax belongs to the Bcl-2 family and is a central regulator of the intrinsic apoptotic pathway. In response to apoptotic stimuli, they are activated and oligomerized on the outer mitochondrial membrane to mediate permeability, which is thought to be a key step in apoptosis. As can be seen in Fig. 7B, Bax expression levels were elevated, and Bcl-2 expression levels were decreased in the brains of SAMP8 mice. According to the correlation analysis, Bax was positively correlated with aging, whereas Bcl-2 was negatively correlated with aging^30^. An increase in Bax levels and decrease in Bcl-2 levels indicate an increase in the level of apoptosis. After MSC-Exo treatment, Bax levels decreased, while Bcl-2 levels increased, suggesting that MSC-Exos may reduce the level of apoptosis in brain tissue to exert an anti-aging effect.

According to studies, SIRT1 is a cellular defense protein that ensures survival by controlling metabolism when there is not enough energy supply. SIRT1 is a crucial molecule in the control of redox states, apoptosis, and life-extending mechanisms. By changing SIRT1 expression, a number of substances and factors can control the level of SIRT1 protein. SIRT1 protein expression declines as a result of aging, and SIRT1 expression decreases with age in mice^31^. SIRT1 has been referred to as a longevity-associated protein that could be used as a potential pharmacological target for extending human lifespan, and it is at the forefront of the battle against cognitive decline, neurodegenerative diseases, and aging. SIRT1 has been reported to negatively regulate the expression of a number of inflammatory senescence-associated secretory phenotype (SASP) factors, including the SASP factor. SIRT1 produces neuronal protection in neurodegenerative disorders and memory impairment, and is crucial for synaptic plasticity and memory retention in neurons^32^. Numerous studies have shown that p53 and p21 have a role in the control of the cell cycle, DNA repair, apoptosis, and other critical biological processes. Cell cycle arrest results from the activation of p53 and p21, which are responsible for replicative and stress-related senescence in cells. Senescent cells release a range of inflammatory proteins, such as SASP, which causes low-grade chronic inflammation and accelerates senescence. Loss of the key anti-aging molecule SIRT1 may be important for accelerating aging^33^. From Fig. 7A we find that, in contrast to SAMR1 mice, SAMP8 mice displayed upregulation of senescence-related signals such p53 and p21 and downregulation of SIRT1 in the hippocampus. These abnormalities were reversed by MSC-Exos.

## Materials and methods

### Chemicals

H_2_O_2_, tamoxifen, and dimethyl sulfoxide (DMSO) were purchased from Sigma Aldrich (St. Louis, MO, USA). Commercial kits for detecting glutathione peroxidase (GSH-Px, No. 20211215), malondialdehyde (MDA, No. 20211214), total superoxide dismutase (T-SOD, No. 20211209), and reactive oxygen species (ROS, No. E004-1-1) were purchased from the Nanjing Jiancheng Institute of Bioengineering (Nanjing, China). Antibodies against Bcl-2, Bax, p53, p21, glial fibrillary acidic protein (GFAP), nuclear factor red lineage 2-related factor 2 (Nrf2), and heme oxygenase-1 (HO-1) were obtained from Proteintech (Wuhan, China), and SIRT1 antibodies were purchased from Abcam (Cambridge, UK). The only additional chemicals and solvents employed in this research were of analytical grade.

### Culture and induction of microglia (BV2)

Microglia (BV2) were purchased from Guangzhou Saiku Biotechnology Co., Ltd. and grown in DMEM/F12 media containing 10% fetal bovine serum (FBS), 1% penicillin/streptomycin, and humidified incubator conditions at 37 °C with 5% CO2. The cells were grown to approximately 70% confluence, induced with 200 μM H_2_O_2_ for 2 h, and then returned to fresh medium and cultured for 3 days to create a model of cellular senescence. The results of senescence induction were judged by β-galactosidase staining, and the senescent cells were stained blue.

### Cell counting kit-8 (CCK-8) assay

To assess cell viability, the CCK-8 kit (C0038; Beyotime, Beijing, China) was applied. Microglia were cultured in 96-well plates at a density of 1 × 10^4^ cells per well, divided into 10 groups, and treated with phosphate-buffered saline (PBS) and 50, 100, 200, 400, and 800 μM H_2_O_2_ for 3 and 6 h (n = 6 samples per group). The medium was removed and a mixture of 10 μL CCK-8 solution with 100 μL DMEM/F12 was added to each well, and the cells were incubated for 2 h. Cell viability was estimated by measuring the optical density of microglia at 450 nm using a microplate reader (Bio-Rad Laboratories Inc., Hercules, CA, USA).

### Reactive oxygen species (ROS) detection

Staining with 2′,7′-dichlorodihydrofluorescein diacetate (DCF) (D6883, Sigma-Aldrich) allowed the detection of H_2_O_2_ and superoxide anions and the measurement of ROS production in BV2 cells. BV2 cells were seeded onto 6-well plates at a density of 3 × 10^5^ cells/well in culture medium (DMEM/F12). After the cells reached 80% confluence, H_2_O_2_ stimulation was performed, followed by incubation with MSC-Exos for 24 h. After the indicated times, the cells were incubated with 10 μM DCF in phenol red-free medium for 30 min at 37 °C. Finally, the cells were washed with pre-warmed PBS and visualized within 1 h using DxFLEX flow cytometry (Beckman Coulter, Brea, CA, USA). Fluorescence intensity was analyzed using Image Pro Plus version 6.0 (Media Cybernetics, Silver Spring, MD, USA). Each experiment was performed with a minimum of five samples, and each assay was performed in triplicate.

### MSC-Exo separation and characterization

Exosomes were extracted from the culture supernatants of fresh or frozen MSCs. The cells were removed by centrifugation at 300×*g* for 10 min. The supernatant was then centrifuged at 2000×*g* for 10 min to remove smaller cell debris and 10,000×*g* for 60 min to remove apoptotic bodies. The exosomes were harvested in a swinging bucket rotor (SW32TI, XPN-100, Beckman Coulter) at 100,000×*g* for 60 min. All centrifugation steps were performed at 4 °C. The cells were resuspended in PBS and stored at − 80 °C. Quantification of the protein content of exosomes was performed using the bicinchoninic acid (BCA) assay (P0010, Beyotime). The expression of the representative exosomal markers TSG101 (1:1000, Abcam), CD81 (1:1000, Abcam), and Alix (1:1000, Abcam) was determined by western blotting. In addition, the morphology and size distribution of exosomes were examined using transmission electron microscopy (TEM) (Hitachi H7650, Tokyo, Japan) and nanoparticle tracking analysis (Malvern Panalytical, Malvern, UK). The protein content of MSC-Exos was normalized to the total protein content and quantified using the BCA method.

### Cell migration assay

The effect of MSC-Exos on BV2 migration was assessed using a scratch assay. Briefly, cells were inoculated in six-well plates using DMEM/F12 medium supplemented with 10% FBS (fetal bovine serum) and 1% penicillin/streptomycin in HYCLONE. To measure cell migration, remove the silica insert after 24 h, wash the resulting gap and fill each well with fresh serum-free medium containing Exos (50 μg/mL). Images of the closed areas were obtained after 0, 24, and 48 h.

$$ {\text{Wound - size reduction }}\left( \% \right) \, = \, \left( {{\text{A}}_{0} - {\text{ A}}_{{\text{t}}} } \right)/{\text{A}}0*{1}00 $$

where A_0_ is the initial wound area, and A_t_ is the wound area 24, 48 h post-wounding.

### Experimental animals and models

Male SAMR1 mice and male SAMP8 mice (weight 20–30 g, age 3.5 months) were purchased from Peking University. The animals used in this study were housed in individual standard cages and maintained in a temperature-controlled room at 21–23 °C on a 12 h light–dark cycle with free access to water and a standard pellet diet. All animal experiments were approved by the Animal Experimentation Ethics Committee of Jilin University and conducted in accordance with internationally accepted guidelines for animal care.

After 3 months of adaptive feeding, 24 SAMP8 mice were divided into two groups of 12 mice each: the senescence model group (SAMP8) and the MSC-derived exosome treatment group (SAMP8-Exo, 10^9^/each, tail vein injection), and treated once a month for a total of three treatments. SAMR1 mice were used as a control group and injected with PBS buffer at the same frequency as the experimental groups. Behavioral experiments were performed at the end of the treatment. All mice were fed a standard pellet diet, and water was freely available.

After the assay, we used the cervical dislocation method to euthanize mice, and blood samples collected from their eyes were used for the measurement of biochemical parameters. Brain tissue was dissected on ice. A portion of the brain was cut longitudinally, fixed in 4% paraformaldehyde, and stained with hematoxylin and eosin (H&E) for pathological studies, while the rest of the brain tissue was homogenized for further experiments. Blood samples and brain tissue were rapidly frozen and stored at − 80 °C until biochemical assays were performed. All experiments were performed in accordance with the Animal Research: Reporting of In Vivo Experiments (ARRIVE) guidelines.

### Behavioral tests

#### Morris water maze test

To assess the spatial learning and memory abilities of SAMP8 and SAMR1 mice, we performed the Morris water maze test (MWM) according to a previously described protocol, with some modifications. The experimental apparatus consisted of a circular tank (120 cm in diameter and 50 cm high) containing water to a depth of 25 cm (22 ± 1 °C) divided into four quadrants (northeast [NE], northwest [NW], southeast [SE], and southwest [SW]) and made opaque by the addition of a black non-toxic dye. A small platform (10 cm in diameter and 22 cm high) was placed in the center of the SE quadrant and submerged at a fixed position (1.5 cm) below the water surface. The MWM program includes navigation training and detection tests. During the learning and memory training period, each mouse was tested four times a day for four consecutive days. Once the mouse was placed in the water maze, if the animal could not find the submerged platform within 60 s, it was manually guided to the platform and left on the platform for 15 s to remember its position. At the end of the training trial, each mouse was tested for 60 s in a detection test without a platform.

#### Contextual fear-conditioning test

The contextual fear conditioning test is a 4-day test. On the first day of the conditioned reflex period, the mice were placed in a conditioned reflex chamber (Med Associates) for 3 min (phase A) and then a sound (2800 Hz and 85 dB) was presented for 30 s (phase B, conditioned reflex stimulus). The last 2 s of the conditioned stimulus was accompanied by 0.7 mA of continuous foot shock (phase C, unconditioned stimulus), followed by resting for 30 s. Phases B and C were repeated once, and the mice were placed back in the cage after 30 s of rest in the reaction chamber. The next day, the contextual memory of the mice was tested for 3 min in the same reaction chamber, without sound or foot shock. On the third day, the sound memory of the mice was tested in another reflex chamber environment with sound, but without foot electroshock. On the fourth day, the environment was changed, and a 3-min environmental memory test was performed to eliminate the effect of the environment on the mice. Fear memory was measured as a percentage of freezing. Freezing was defined as the percentage of time with a 5 s interval of complete absence of movement (except breathing).

### Histomorphological assessment

Fresh brain biopsy specimens were collected from mice, fixed in 4% paraformaldehyde (pH 7.4) for 4 h at 4 °C, incubated overnight at 4 °C in 100 mM sodium phosphate buffer (pH 7.4) containing 30% sucrose, and embedded in paraffin. Sections (4 μm thick) were then stained with hematoxylin and eosin (H&E) and Nissl according to standard procedures and examined under a microscope.

### Immunohistochemistry

Immunohistochemistry was used to detect the expression of GFAP in brain tissue. Paraffin-embedded tissue was cut into 4-μm sections, mounted on glass slides, and stained using indirect immunooxidase. Paraffin sections were baked at 65 °C for 24 h, then rinsed three times with PBS for 5 min each. The fully washed fraction was placed in ethylenediaminetetraacetic acid buffer and heated to boiling in a microwave oven. This step was repeated four times for 6 min each. After natural cooling, the sections were washed three times with PBS for 3 min each. The sections were incubated with 3% H_2_O_2_ for 30 min at room temperature, washed three times with PBS, and incubated with 5% bovine serum albumin for 20 min. Next, the sections were incubated overnight at 4 °C with 50 μL of diluted anti-GFAP antibody and then incubated for 50 min at 4 °C with 50–100 μL of goat anti-rabbit secondary antibody. These fractions were washed three times with PBS, incubated in 3,3'-diaminobenzoic acid, and washed with distilled water. Image Pro Plus version 6.0 software (Media Cybernetics) was used to analyze the positively stained areas. Strong positive staining of the cell membrane or cytoplasm of neurons was brown, and the staining intensity indicated GFAP protein expression levels.

### Biochemical analysis

T-SOD (U/mg protein), GSH-Px (U/mg protein), and MDA (nmol/mg protein) levels in whole brain tissue were measured in the pharmacology department using commercially available kits (built in Nanjing) and a multifunctional microplate assay reader (Infinite® 200 PRO, Tecan, Männedorf, Switzerland). All biochemical analyses were performed in accordance with the manufacturer's instructions.

### Western blot analysis

Brain tissue was lysed with radioimmunoprecipitation assay (RIPA) lysis buffer to extract cellular proteins. Tissue homogenates were collected by centrifugation at 12,000 rpm for 30 min at 4 °C, and the supernatant was extracted for concentration determination using a Beyoncé BCA kit. Equal amounts of protein (50 μg) were separated by 10% sodium dodecyl sulfate–polyacrylamide gel electrophoresis (SDS–PAGE) and then transferred to nitrocellulose membranes, which were blocked with 5% skim milk powder (dissolved in Tris-buffered saline with Tween-20 [TBST]) for 1 h at room temperature. The membranes were incubated overnight at 4 °C with various antibodies diluted at 1:1000, washed, then incubated with HRP-labeled goat anti-rabbit IgG (1:10,000) or anti-mouse IgG (1:10,000) for 1 h, followed by three washes with TBST. Finally, images of the target proteins were collected and analyzed using a two-color infrared laser imaging system (Licor Odyssey, USA).

### Statistical analysis

Data are expressed as mean ± standard deviation (SD). Multiple comparisons were analyzed using one-way analysis of variance (ANOVA) by Least Significant Difference (LSD) test, and repeated measures ANOVA was carried out to analyze the differences in learning curves and traveling distance among the four groups, followed by Bonferroni post-hoc test using SPSS 19.0.0 (IBM, Armonk, NY, USA) and GraphPad Prism 7 software (GraphPad, San Diego, CA, USA). *P* values of less than 0.05 were considered statistically significant (Supplementary Information S1).

### Supplementary Information

Supplementary Information.

## Data Availability

The data that support the findings of this study are available from the corresponding author, [Jilin University Sino-Japanese Friendship Hospital], upon reasonable request.
