# Supplementary material for: Exosomes secreted by mesenchymal stem cells delay brain aging by upregulating SIRT1 expression
Source: Sci Rep. 2023 Aug 14;13:13213. doi: 10.1038/s41598-023-40543-5 (PMC10425430; doi:10.1038/s41598-023-40543-5)
Supplement: Supplementary file 1 — Supplementary Information. [file 41598_2023_40543_MOESM1_ESM.pdf]

1 . BAX（red）GAPDH（green）

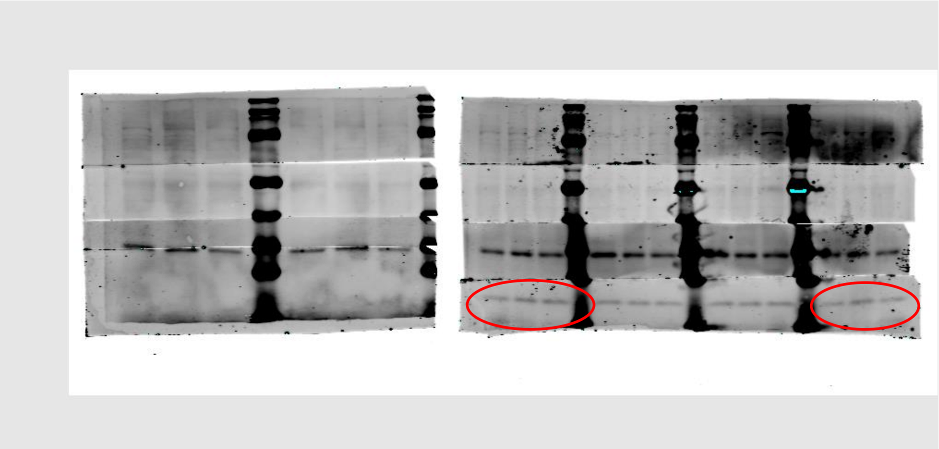

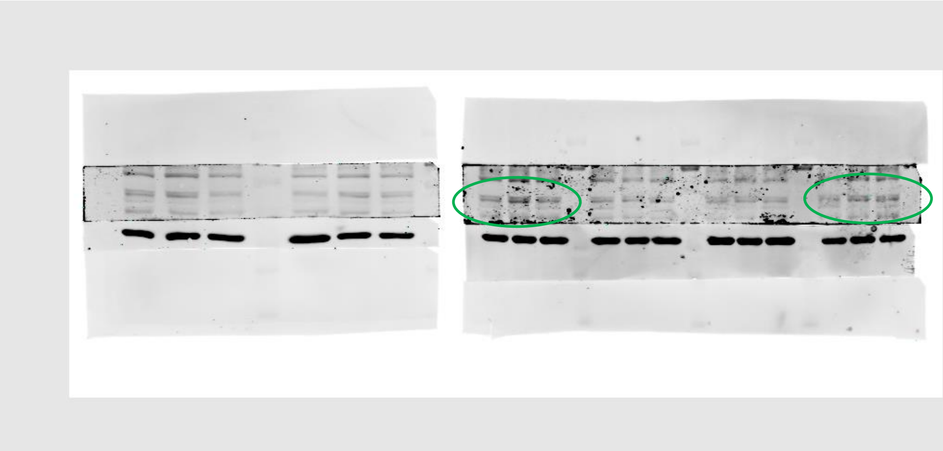

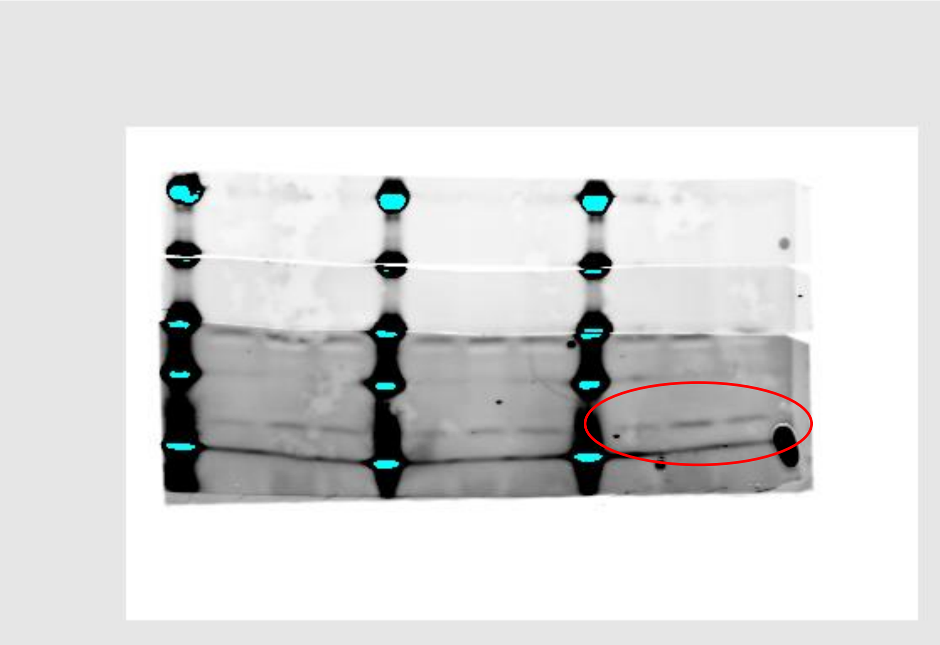

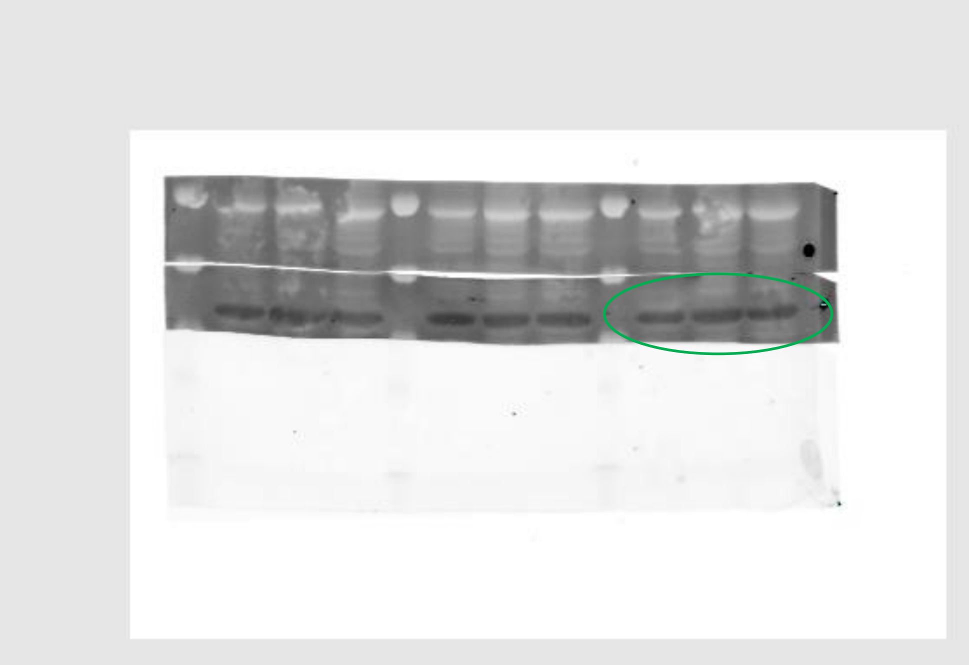

2.BCL-2(red) GADPH(green) β-actin(yellow)

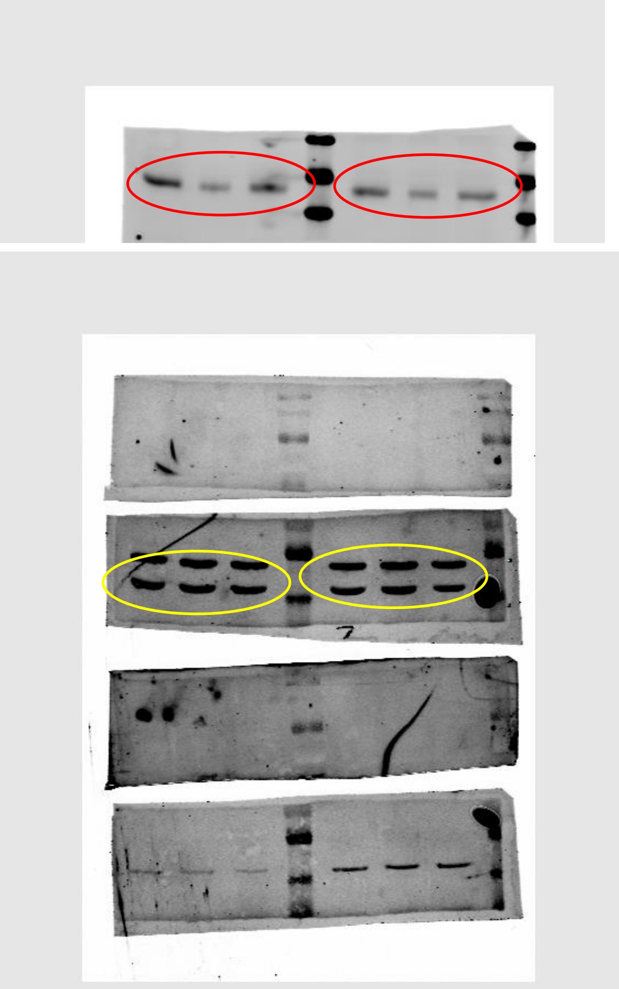

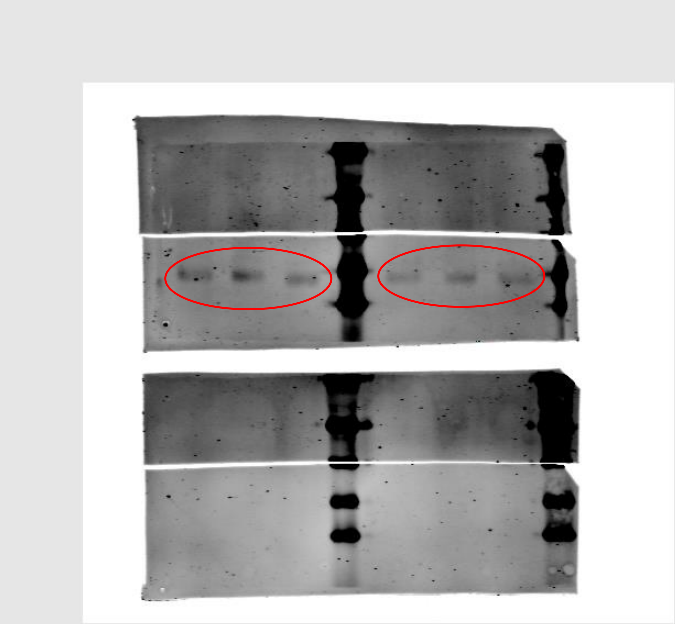

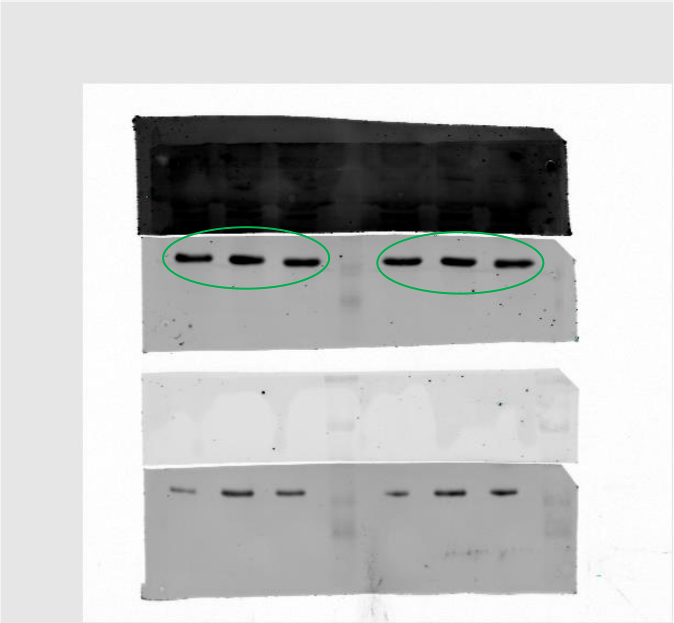

3. HO-1(red) GADPH(green) β-actin(yellow)

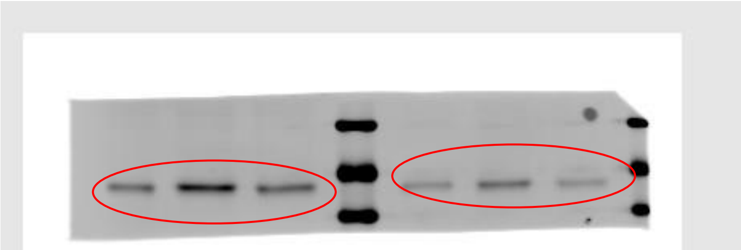

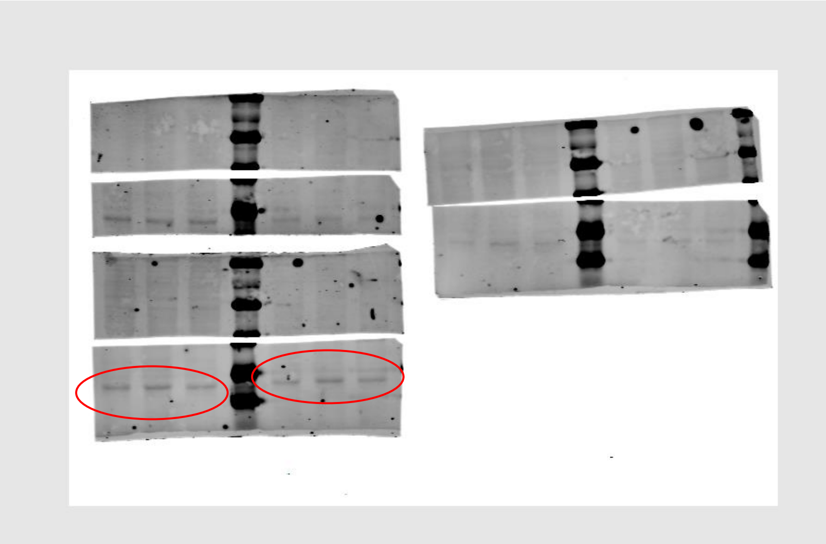

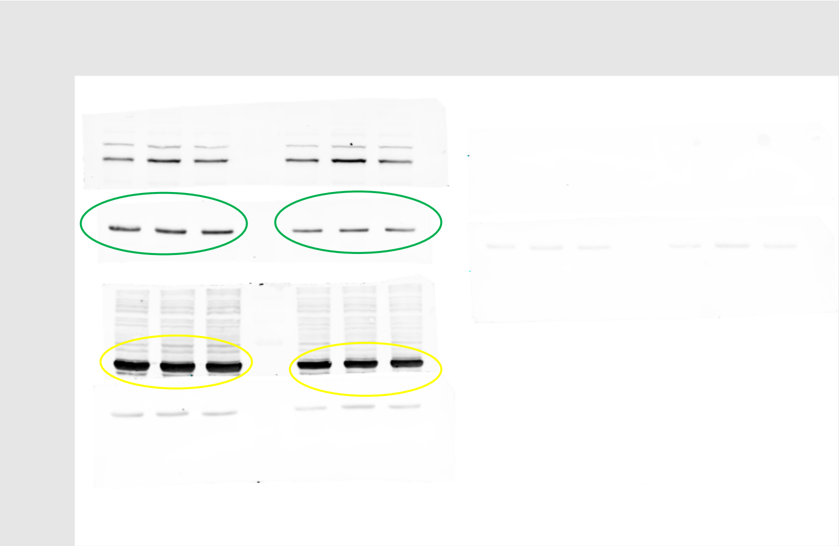

4.NRF2(red) GADPH(green)

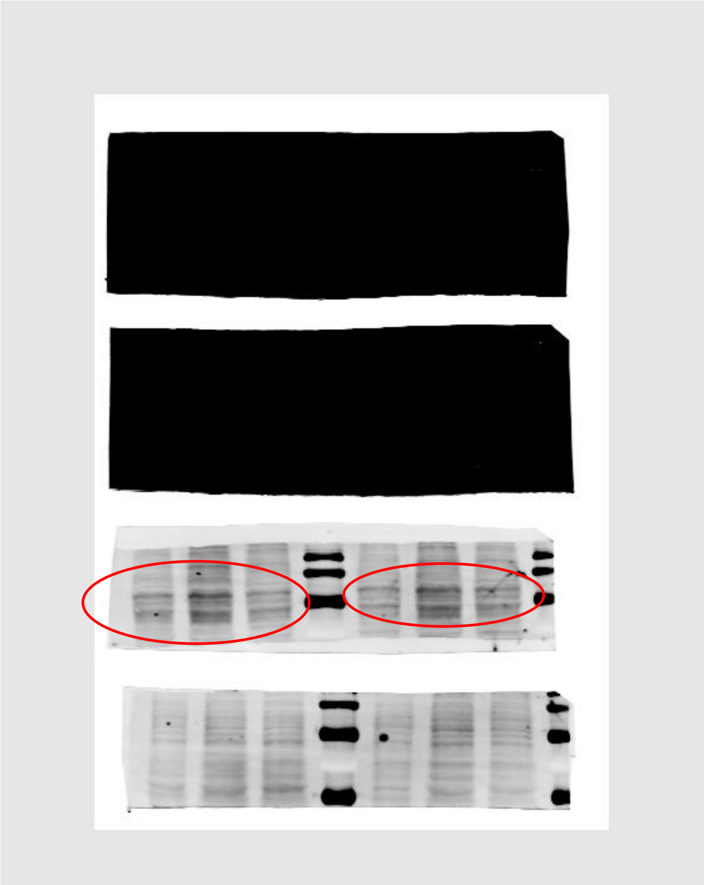

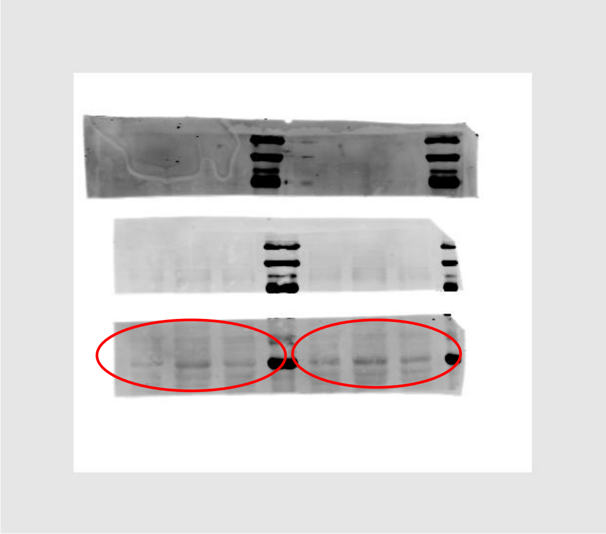

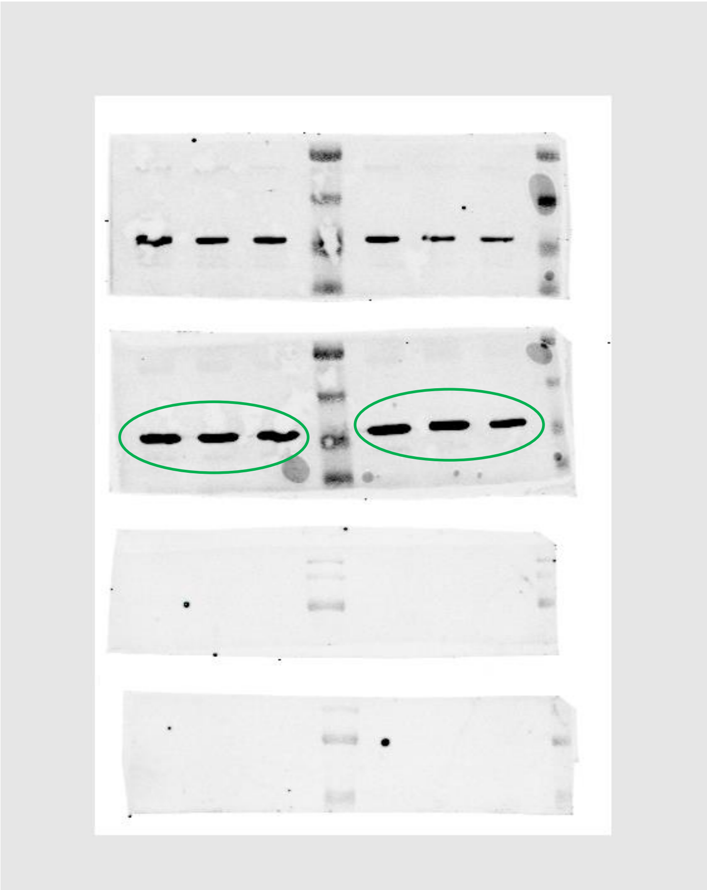

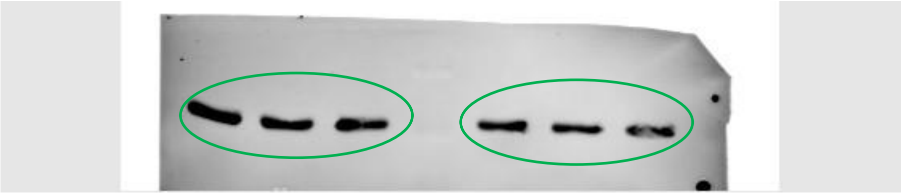

5.p21(red) GADPH(green)

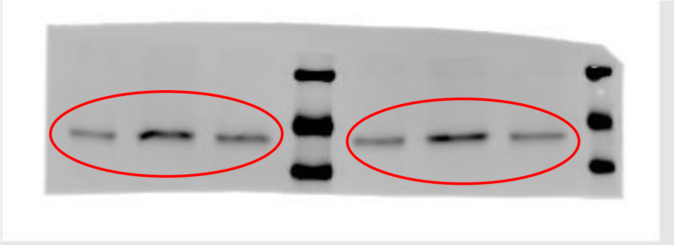

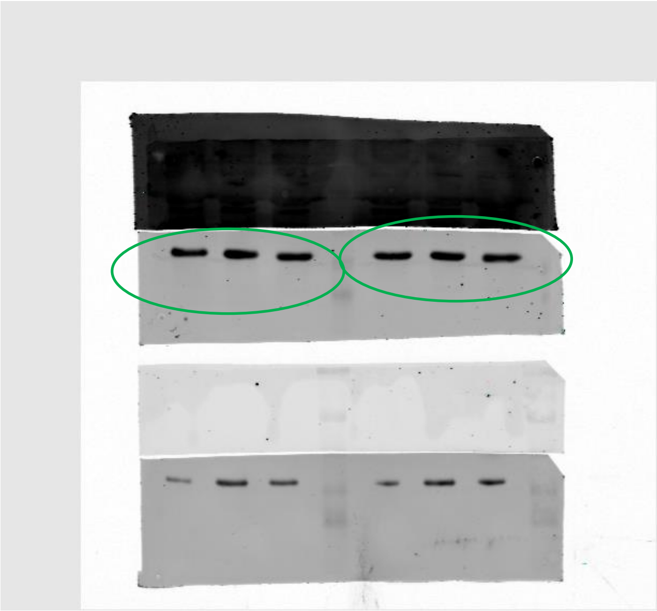

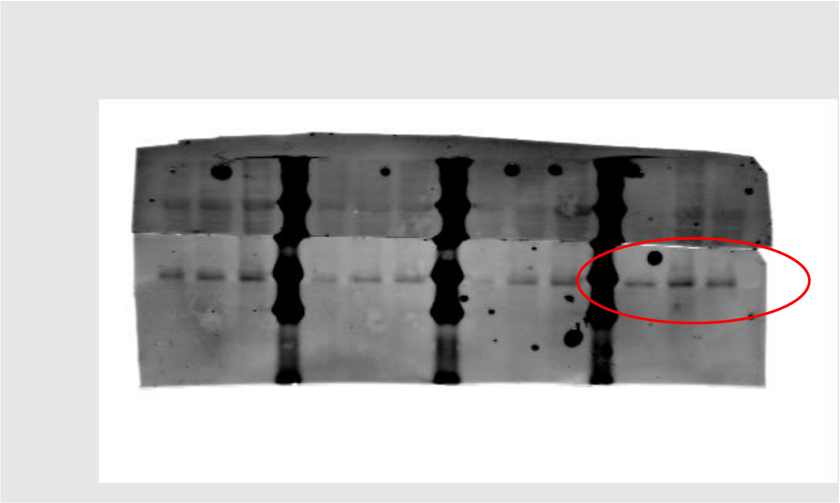

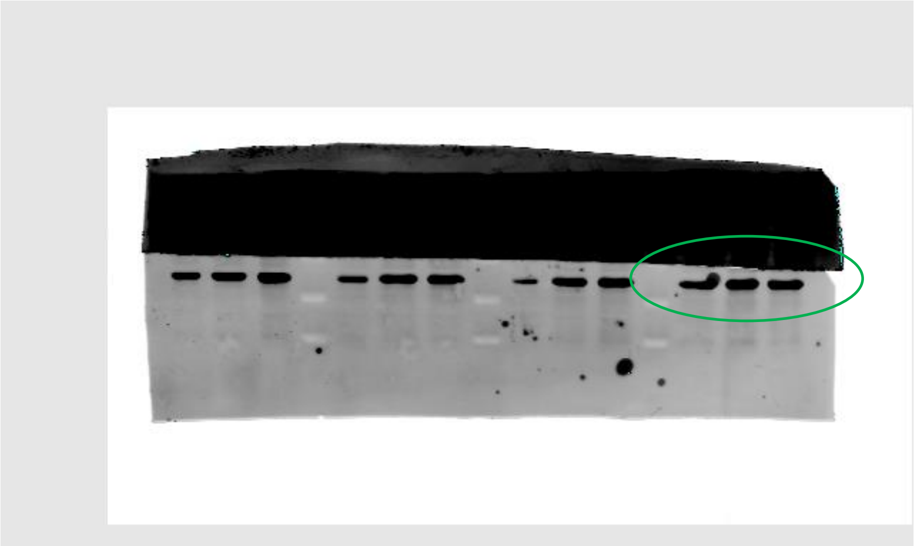

6.p53(red) GADPH(green)

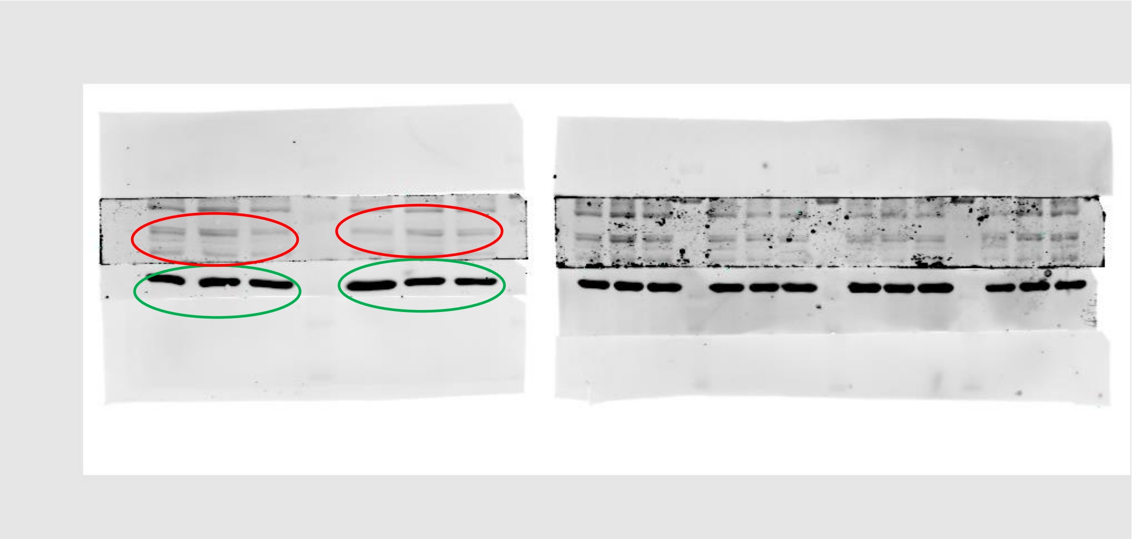

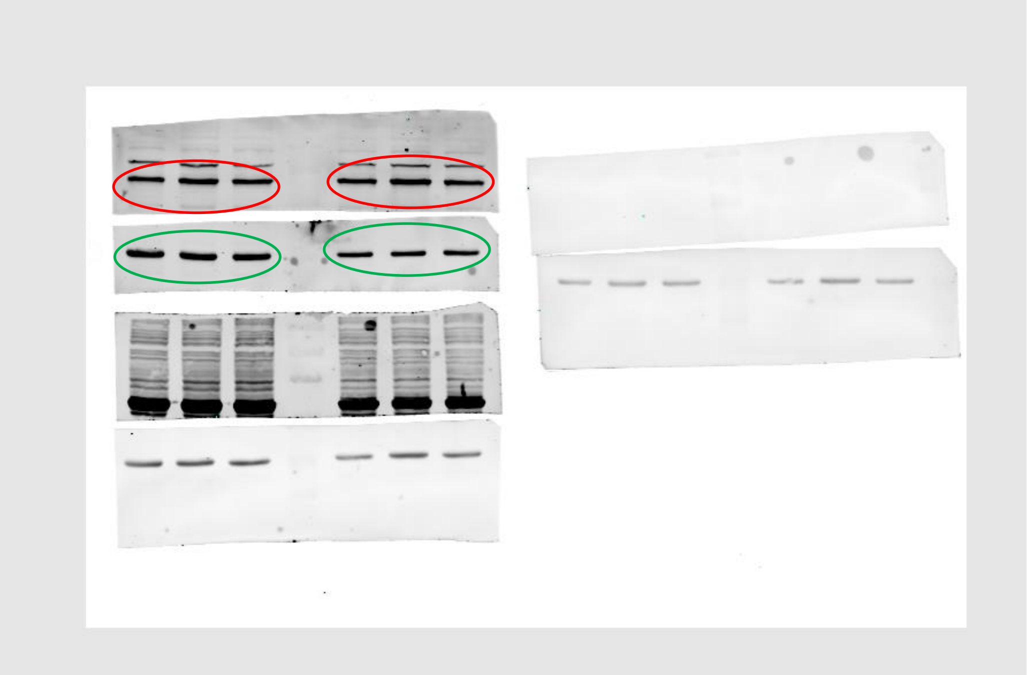

7.SIRT1(red) GADPH(green)

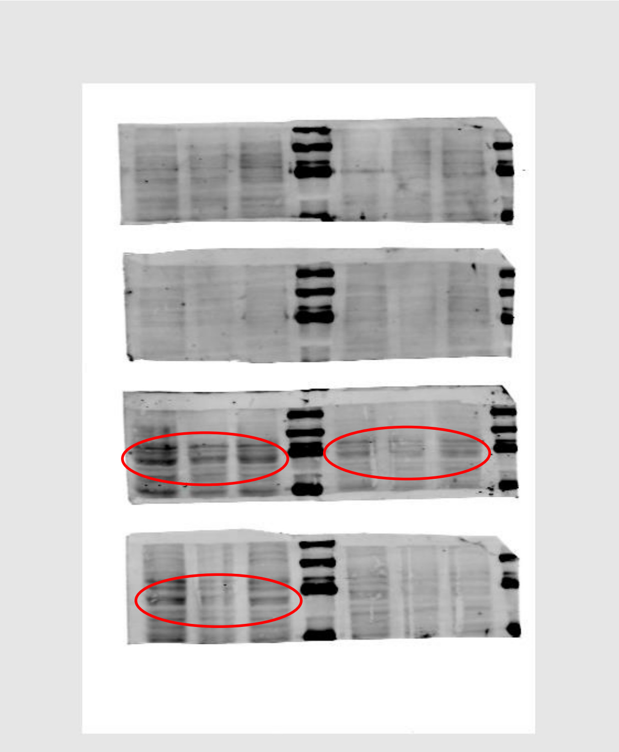

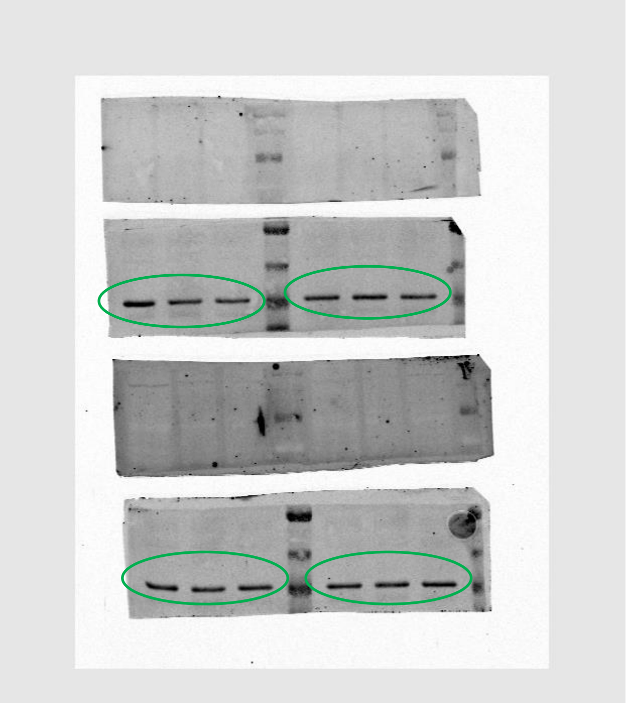

ALIX（red）

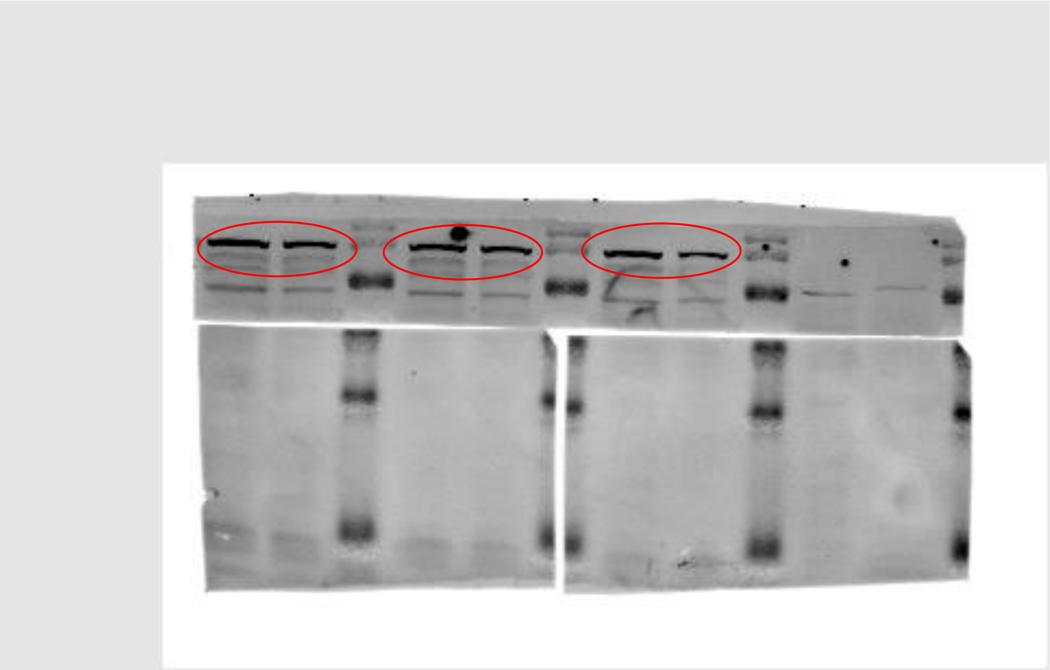

TSG101（blue）

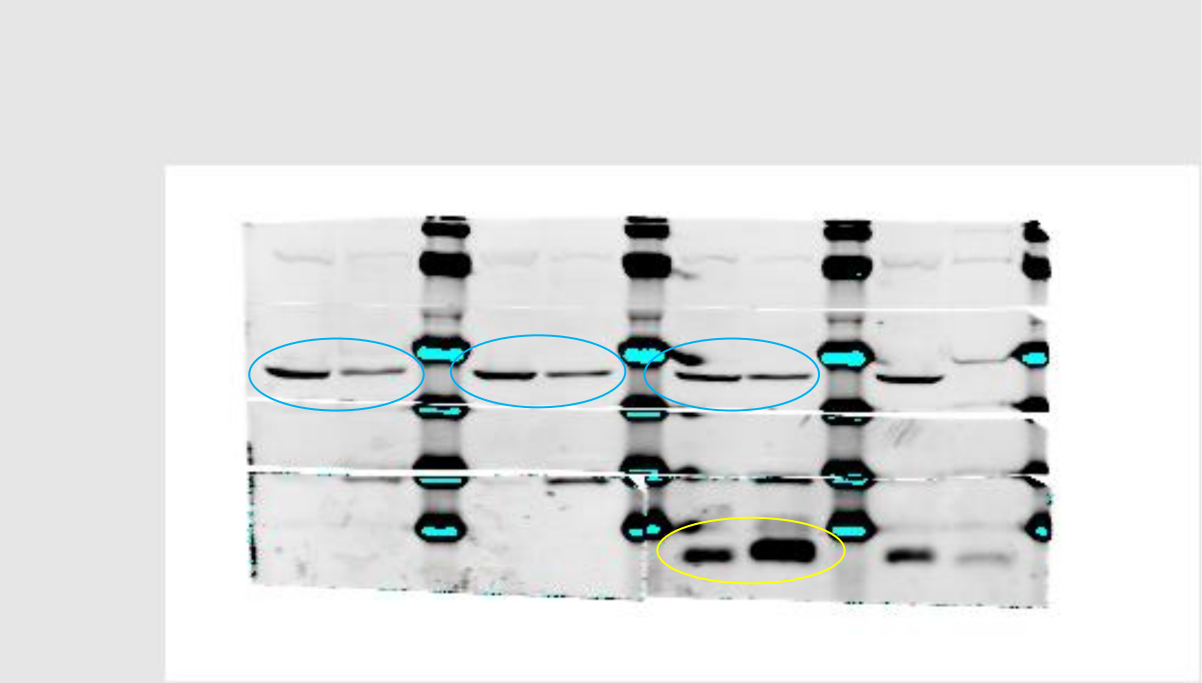

CD9（yellow）

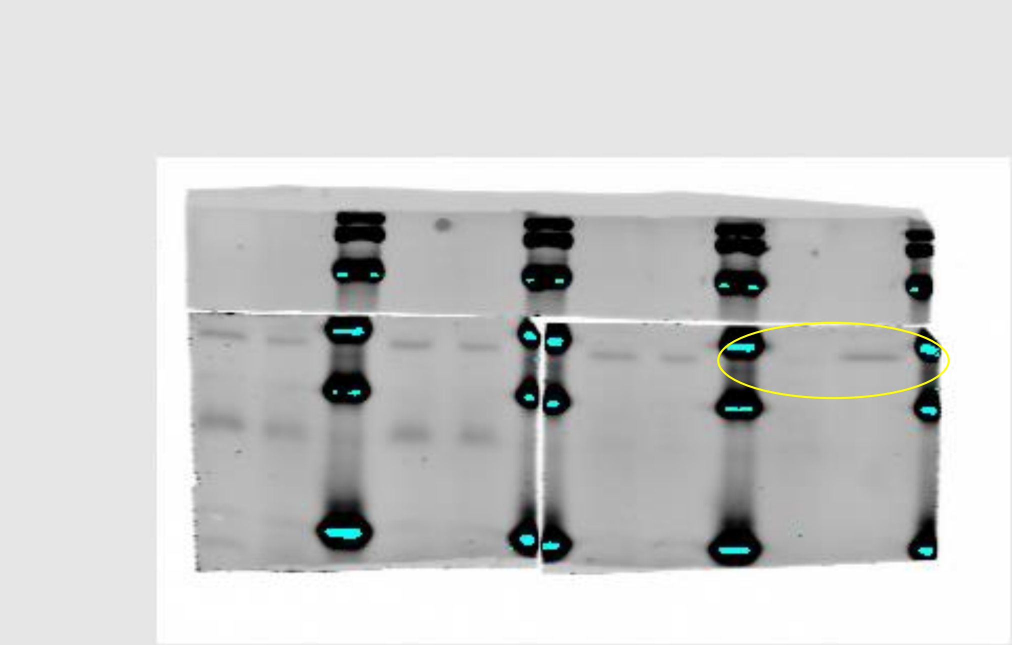

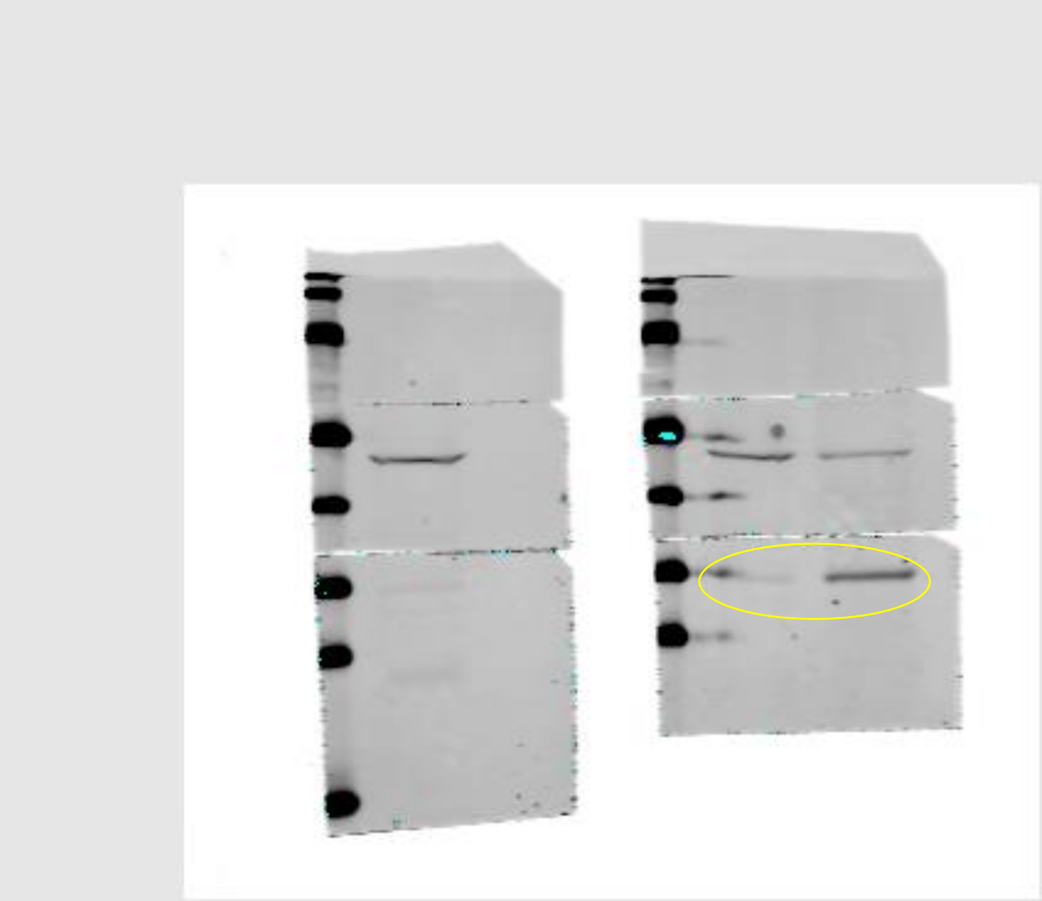

EXO-GAPDH （green）

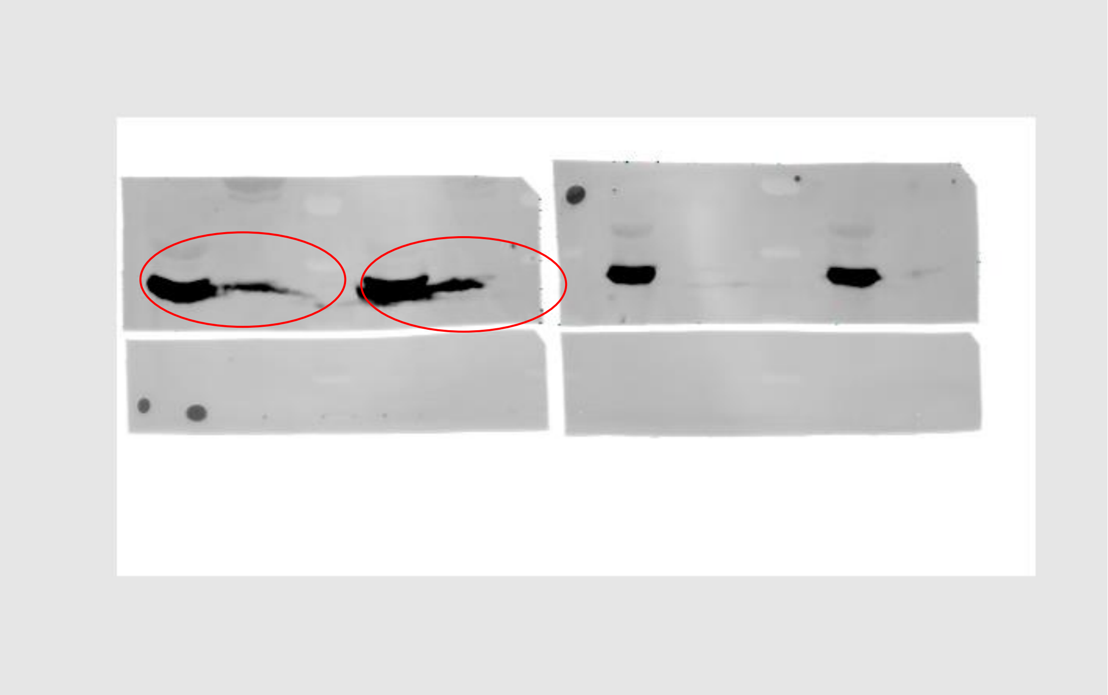

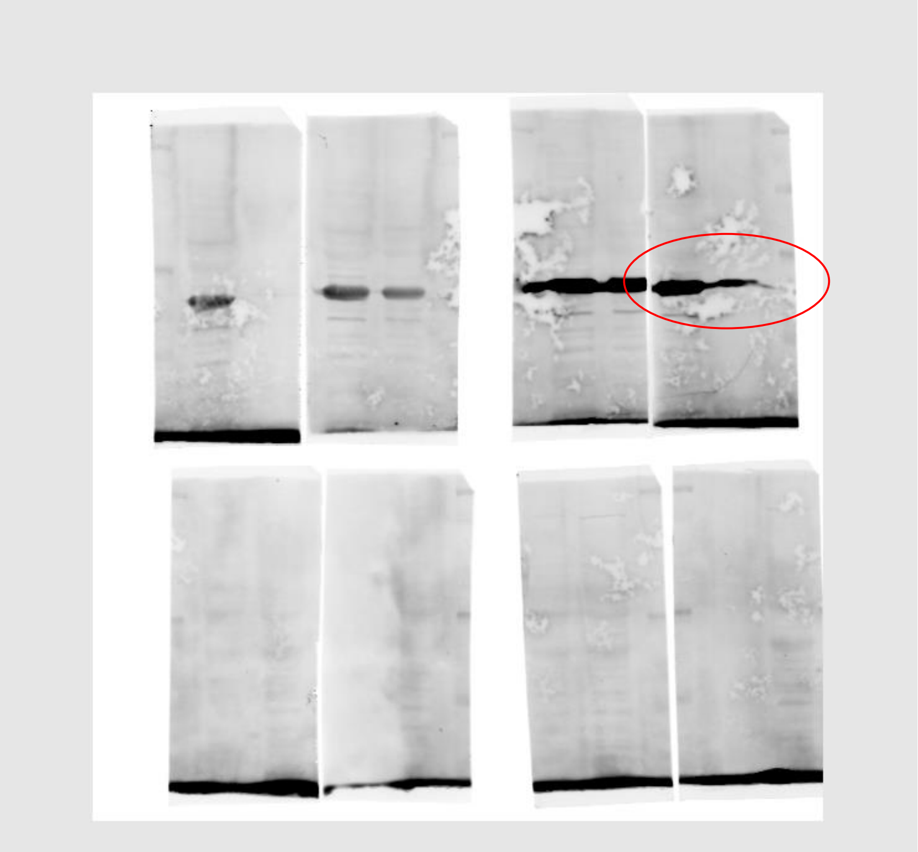

Original of Figure 6E – GAPDH（red）

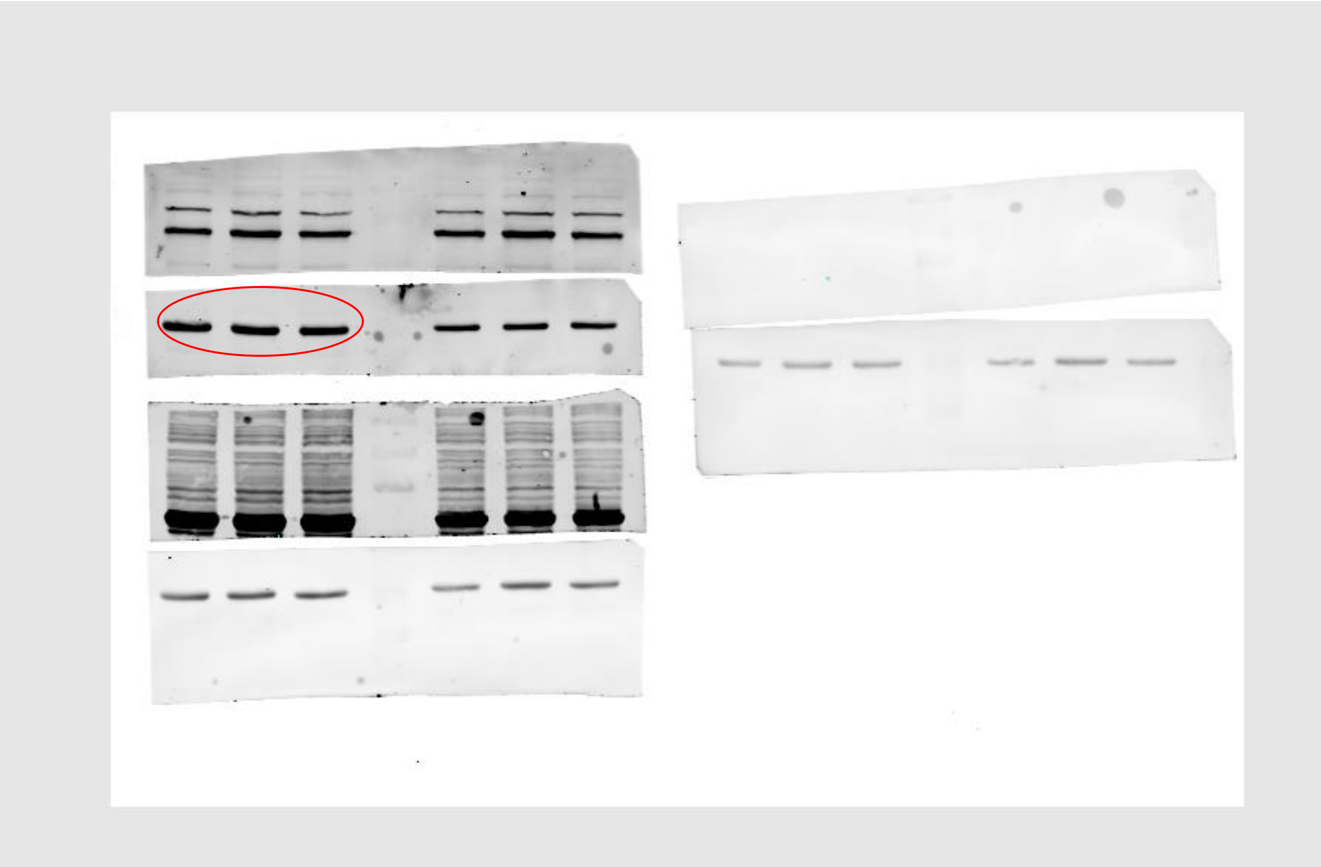

Dear Editor, I apologize for mixing up the GAPDH for Figure 6E. I have reorganized the figure and inserted the new GAPDH into Figure 6E.
